# Characterizing the American Upper Paleolithic

**DOI:** 10.1126/sciadv.ady9545

**Published:** 2025-10-22

**Authors:** David B. Madsen, Loren G. Davis, Thomas J. Williams, Masami Izuho, Fumie Iizuka

**Affiliations:** ^1^ Department of Anthropology, University of Nevada-Reno, Reno, NV, USA.; ^2^ Department of Anthropology, Oregon State University, Corvallis, OR, USA.; ^3^ Spokane Tribe of Indians Preservation Program, Wellpinit, WA, USA.; ^4^ Department of History and Archaeology, Tokyo Metropolitan University, Tokyo, Tokyo, Japan.; ^5^ Department of Anthropology, University of Wisconsin-Madison, Madison, WI, USA.; ^6^ Center for Northeast Asian Studies, Tohoku University, Sendai, Miyagi, Japan.

## Abstract

In North America, there are enough sites with relatively large tool assemblages predating ~13,500 calibrated years before the present (cal yr B.P.) to allow assessment of the underlying characteristics of their shared lithic tradition. Their shared technological features involve the use of dual core-and-blade and biface technologies similar to those in the Northeast Asian Late Upper Paleolithic. These dual approaches were often merged to produce small projectile points, including stemmed point forms using an elliptical cross-sectional ogive design. Similar dual lithic technologies are found in assemblages in northern Japan dating to ~20,000 cal yr B.P. We suggest a group with a similar lithic technology became isolated somewhere in the vicinity of the Paleo-Sakhalin-Hokkaido-Kuril region, developing genetically into ancestral American populations. Between ~22,000 and ~18,000 cal yr B.P., a subset of this population migrated along the southern Beringian and Northwest coasts into the Americas. By ~16,000 to ~15,000 cal yr B.P., they had become widely dispersed across North America.

## Teaser

Tool similarities link Late Pleistocene American and Northeast Asian lithic traditions.

## INTRODUCTION

In 2013, Collins *et al.* (*1*) defined seven cultural complexes or “patterns” in the Americas predating the Paleoindian period, which begins with the Clovis Paleoindian Tradition (CPT) currently estimated to have begun sometime between ~13,500 and ~13,000 calibrated years before the present (cal yr B.P.) (*2*, *3*). These patterns helped to demonstrate that there was a wide array of lithic complexes found in the New World dating to the ~20,000 to ~13,500 cal yr B.P. period, but what was not clear is that most of these patterns share a broad technological similarity, linking them together into what we refer to as the American Upper Paleolithic (AUP), following Williams and Madsen (*4*) and Davis and Madsen (*5*). Despite heated late 18th century debates about the appropriateness of using “Upper Paleolithic” in discussing early New World lithic technologies (*6*), we think that its use is critical in linking the origins of these early technologies to the rest of the world’s archeological sequences.

In large measure, these complexes are based on the use of dual core-and-blade and biface technologies similar to those found in the Late Upper Paleolithic (LUP) of Northeast Asia, indeed, most of Eurasia. In the core and blade technological system, flat-faced, wedge-shaped, and occasionally Levallois-like and conical cores are used to produce elongate blades and/or blade flakes, which constitute one of the primary tool forms used by the initial occupants of North America. These elongate, mostly prismatic, blade flakes were often used as cutting tools without modification but were also retouched into a variety of other tools such as scrapers, drills, and projectile points. In the parallel biface technology, on the other hand, collateral flaking was used to produce proportionally flaked bifaces, including projectile points, with many of the removal flakes used expediently as modified flakes. These dual technological approaches were often merged to produce small projectile points, which vary in morphological form from site to site. Stemmed, lanceolate, and triangular forms were produced either by direct bifacial reduction or by bifacially reducing elongate blades produced from blade cores. Here, we review the lithic technologies used at a suite of sites dating to the AUP period to identify cross-cutting similarities and then compare the shared technological traditions to those found in Northeast Asia dating to and just before the AUP period.

## BACKGROUND

**Fig. 1.**
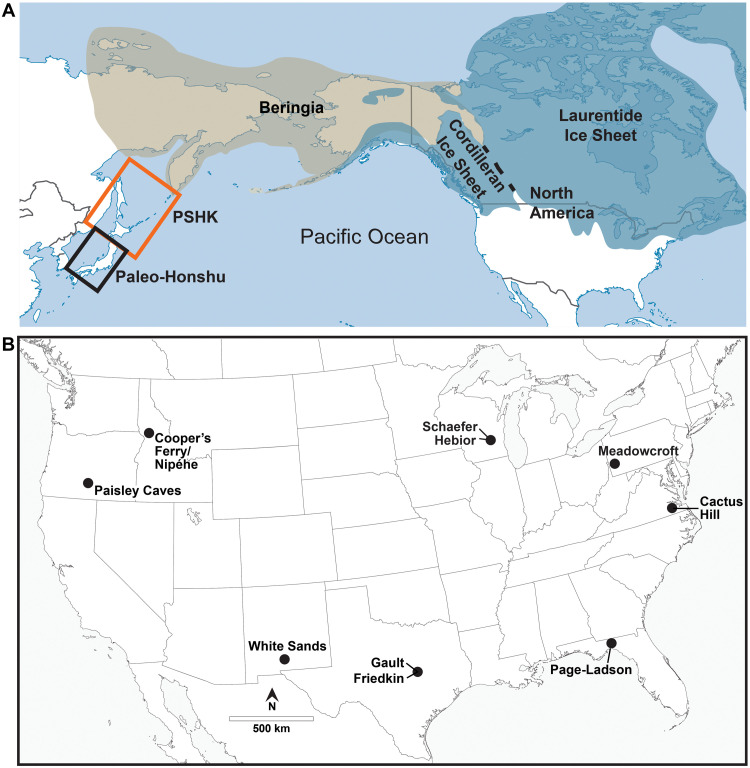
Map of locations and sites. Map showing the locations of major physiographic regions discussed in the text (**A**). Map showing the location of AUP sites in North America discussed in the text (**B**).

The CPT represents clear evidence for a human presence in North America by ~13,500 cal yr B.P. (*2*, *3*). However, the origin and form of the archeological record that predates Clovis remain subjects of ongoing debate (*4*, *5*, *7*–*9*). While it is reasonable to infer that populations must have been present before the emergence of Clovis technology, there is less consensus regarding how such earlier occupations should be identified archeologically. Nevertheless, a number of sites have been proposed as evidence for a pre-Clovis presence in the Americas, with reported ages ranging from ~20,000 to ~13,200 cal yr B.P. and possibly even as early as ~23,000 cal yr B.P., e.g., (*10*, *11*). This latter age is derived from the dating of human footprints at White Sands, New Mexico, e.g., (*12*), but it has been suggested that the associated radiocarbon age estimates may be 7000 to 3000 years too old due to the uptake of older carbon in the dated plant material, e.g., (*13*–*15*). Regardless of their ultimate age, the White Sands footprints, dating to before ~20,000 cal yr B.P. or to “only” ~16,000 cal yr B.P., would still represent some of the oldest evidence of humans in the Americas and would be contemporaneous with the lithic assemblages we discuss here. If the older age is confirmed, then it would place the age of the initial occupation of lower North America well into the last glacial period. Not all of the sites claimed to hold pre-Clovis evidence are widely accepted, and to be conservative, we have identified only 10 where the stratigraphic and chronological relationships with demonstrable human artifacts are robust enough to provide strong evidence for pre-CPT occupations (Fig. 1) (*4*, *16*). Even the validity of these 10 sites has been hotly debated between adherents of a “Clovis First” model for the initial occupations of the Americas and those who favor a “Pre-Clovis” model. We reference some of these pros and cons in the Supplementary Materials as a guide to these debates. However, many of the AUP lithic assemblages we include come from stratified sites, which also contain post-AUP components. These overlying assemblages contain diagnostic artifacts associated with age estimates corresponding to the accepted time ranges for those diagnostics, suggesting that the AUP chronologies are also largely correct. That is, the AUP assemblages stratigraphically underlie cultural deposits of Clovis age and younger and are therefore older.

Only five of the 10 sites have reported lithic assemblages large enough to allow a reasonable assessment of the basic shared AUP lithic technology. These sites are Cactus Hill in Virginia, Meadowcroft Rockshelter in Pennsylvania, the Gault and Debra L. Friedkin sites in Texas, and Cooper’s Ferry/Nipéhe in Idaho (Fig. 1). The Friedkin site, located near the Gault site in central Texas, has a large lithic assemblage of well more than 100,000 artifacts dating to the AUP period, but analysis of the assemblage is not yet complete, and we provide only a limited description of its AUP component. As we noted, the White Sands footprint site is important chronologically, but no associated lithic materials have been identified as yet. Sites such as the Schaefer and Hebior mammoth sites in Wisconsin, Page-Ladson in Florida, and Paisley Caves in Oregon also appear to provide reliable evidence of pre–~13,500 cal yr B.P. occupations but are less useful in defining similarities and differences in basic lithic technology due to the paucity of lithic material they contain. However, their limited lithic collections include the combined blade or blade flake and biface production technologies characteristic of AUP assemblages and/or bifacially produced stemmed points and are pertinent as complementary or supporting sites. While this sample size of sites with notable lithic assemblages is smaller than the total number of sites bearing AUP cultural evidence, it is sufficiently consistent that it provides a preliminary picture of the AUP’s archeological record. We provide brief descriptive summaries of these sites in the Supplementary Materials.

We restrict our comparison of AUP lithic complexes to those from North American sites located south of the southern limits of the Cordilleran and Laurentide ice sheets. In South America, there are relatively few sites with large lithic assemblages predating ~13,500 cal yr B.P. Similarly, we do not consider the earliest lithic assemblages from eastern Beringia that date from ~14,500 to ~13,000 cal yr B.P. and include wedge-shaped microblade technology, microblade and flake tools, and teardrop-shaped projectile points made on thin flakes and preforms (*17*, *18*) because it is possible, if not likely, that the lithic assemblages from these sites are associated with a later technological tradition that differs from the one from which the North American AUP assemblages seem to be derived.

### Principal American Upper Paleolithic assemblages

#### Cactus Hill

Cactus Hill is a stratified dune site adjacent the Nottoway River on the coastal plain of southern Virginia (Fig. 1), e.g., (*19*) (see Supplementary Materials). Laminated sand layers at the site contain early Archaic, Clovis, and AUP deposits separated by 10 to 20 cm of culturally sterile sand (*20*). Chronology at the site suggests that the initial occupation of Cactus Hill was ~18,000 cal yr B.P. or earlier. While there are several dating issues, which contribute to uncertainty about this early age, the relative stratigraphic separation between the AUP and CPT horizons and the differences in technological characteristics between the lithic materials in the two horizons make it clear that the lowest cultural deposits date to some time before the CPT era.

##### Cactus Hill AUP lithic technology

**Fig. 2.**
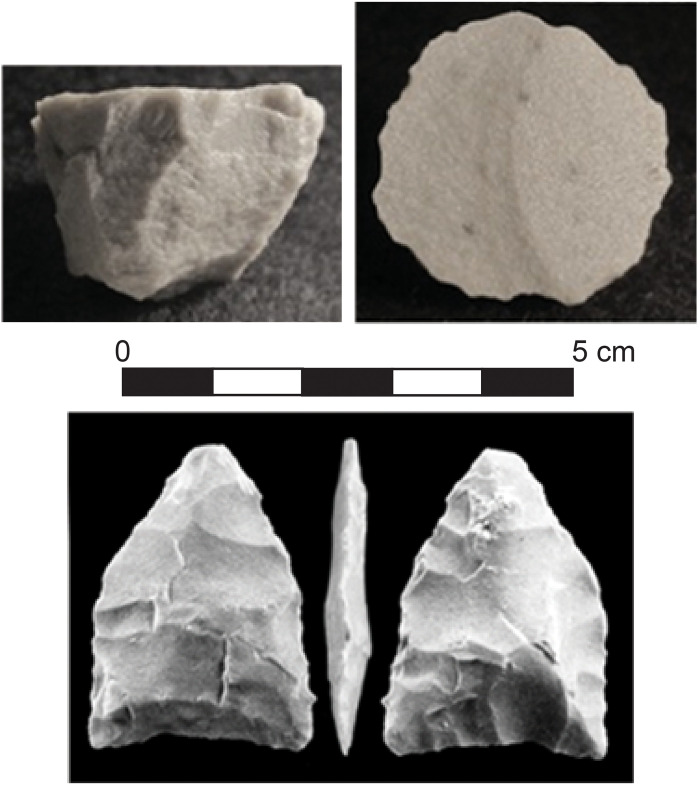
Core and projectile point from Cactus Hill. Typical quartzite blade core from the Cactus Hill AUP assemblage (above). One of two small triangular points from the AUP assemblage at Cactus Hill produced from collateral bifacial flaking (below). The point is 35.5 mm from base to tip. From (*19*); used with permission.

McAvoy and McAvoy (*19*) identify Early and Late Pre-Clovis subperiods from excavated artifacts found at the Cactus Hill site (these assemblages are combined in Table 1). The Early Pre-Clovis subperiod includes polyhedral blade cores and core blades (Fig. 2). The Late Pre-Clovis subperiod is primarily represented by polyhedral blade cores, blades, and bifacial projectile points but also includes linear blade flakes, flake tools, unifacial end scrapers and side scrapers, tabular and irregular shaped grinding and abrading stones, and bifacial preforms [(*19*); figures 5.96 to 5.99]. The two small triangular points produced using bifacial collateral flaking are morphologically very similar to the “Miller” point from Meadowcroft Rockshelter but lack basal edge grinding. The authors also suggest that the recovery of a fragmentary burned bone “small projectile point tip” and abrading stones may signal the presence of an early bone tool industry at the Cactus Hill site (*19*).

**Table 1.** Tool types in five AUP lithic assemblages. Friedkin site tool quantities are based on data reported in (*29*) with the additional 12 projectile points reported in (*30*). Hence, these Friedkin site tool quantities reflect preliminary minimum values, as the final artifact analysis is incomplete.

Artifact						
Category	Meadowcroft	Cactus Hill	Gault	Cooper’s Ferry	Debra L. Friedkin	Totals
Abrader	–	4	1		–	6
Bifaces	2	1	38	6	1	59
Blades/bladelets	13	101	86	6	19	226
Burins	–	10	10	2	1	23
Blade cores	–	8	6	2	2?	18
Discoidal cores	2	–	4	–	1	7
Levallois-like cores	–	–	–	2	–	2
Graver/perforators	2	–	33	–	1	36
Modified flakes	2	5	250	21	17	295
Projectile points	1	2	10	34	12	59
Retouched blade/flake tools	4	5	5	–	5	19
Unifaces	–	?	14	2	–	18
Scrapers	–	5	7	–	13	25
Tool subtotal	26	141	464	76	83	790
Debitage	298	~670	27,285	1,206	15,490	44,949

#### Meadowcroft Rockshelter

Meadowcroft is a well-stratified rockshelter along a tributary of the Ohio River in western Pennsylvania containing an archeological record spanning the entire precontact period (Fig. 1) (see Supplementary Materials). The chronology for 11 stratigraphic units is controlled by more than 50 radiocarbon dates. These are in the correct stratigraphic order and are associated with diagnostic artifacts of the appropriate periods. The earliest AUP occupations are found in lower Stratum IIa, which dates to “…sometime between 13,955 and 14,555 radiocarbon years ago” (*21*).

##### Meadowcroft AUP lithic technology

The AUP lithic assemblage at Meadowcroft is somewhat smaller than the other primary assemblages discussed here, consisting of 26 worked tools and 298 unused pieces of debitage. It is, however, sufficiently large that it provides an adequate assessment of the overall technology found at the site during the AUP period. These numbers differ somewhat from those presented in some of the Meadowcroft publications, e.g., (*22*, *23*), primarily because we do not include the lithic assemblages from middle and upper Stratum IIa. The numbers represented here are a product of the most recent analysis of the Stratum IIa lithic assemblage. The assemblage is derived from the lower portion of Stratum IIa, beginning 40 cm below the surface of the stratum and consists of a mix of bifacial tool production and core-and-blade technologies.

**Fig. 3.**
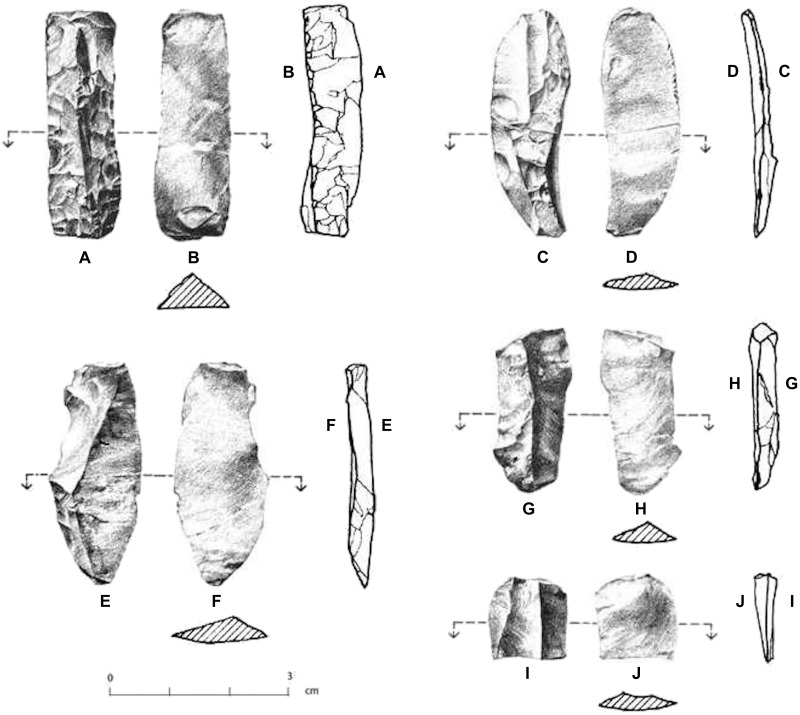
Prismatic blade specimens from the AUP component at Meadowcroft Rockshelter. “A” and “B” are the dorsal and ventral surfaces of a crested blade, which appears to be a product of prismatic blade core preparation. Other prismatic blades are oriented to show their dorsal (C, F, G, and I) and ventral (D, F, H, J) surfaces. Reproduced from Adovasio *et al.* (*23*) (copyright 1999, SAGE Publications; reprinted by permission of SAGE Publications; RightsLink license no. 6090510866501).

The 13 blade or blade fragments in the Meadowcroft AUP assemblage are “…small, prismatic blades that were detached from small, prepared cores” (*23*). No blade cores were recovered during the excavation, but the nearby undated, but possibly contemporaneous, Krajacic site contains both similar blade forms and small, cylindrical polyhedral cores, which “…precisely parallel the core reduction strategy…” posited for the AUP blades at Meadowcroft (*23*). The blade collection includes a crested blade or lame à crête, which may possibly indicate that prismatic blade core production is also represented in the Meadowcroft AUP assemblages. Several of the blades have negative flake scars on their dorsal surfaces, indicating prior removals of other blades not represented in the recovered assemblage (Fig. 3).

The AUP bifacial technology at the site appears geared to the production of bifacial cutting tools and flakes. Two discoidal core fragments appear related to flake production. The limited collection of bifaces and bifacially worked tools includes a unique form that Adovasio *et al.* (*24*) call “Mungai Knives.” These consist of “…bilaterally retouched rhomboidal flakes…” worked only along the flake margins. One complete biface found on a “living surface” in lower Stratum IIa was identified as a resharpened lanceolate projectile point termed the “Miller Point” (*24*). This was produced using collateral flaking for shaping and retouch rather than the thinning method used to produce CPT lanceolates. The point has a basally thinned, edge ground, and ~18-mm-long haft, which tapers slightly from ~23 mm at its juncture with the point blade to ~21 mm at the square base. Although the blade has been resharpened and its original morphology is unknown, the point does not appear to have been shouldered.

#### Gault

Gault is an open site located along a small spring-fed stream on the ecotone between the central Texas hill country of the Edwards Plateau and the Blackland Prairie on the adjacent coastal plains (Fig. 1), e.g., (*25*) (See Supplementary Materials). More than 150,000 AUP artifacts, termed the “Gault Assemblage,” were recovered from the lowest two stratigraphic units (units 1 and 2) above bedrock in Area 15 of the site. These were separated from a similar number of CPT artifacts in unit 4 by a 10- to 15-cm-thick zone of markedly reduced lithic debris. The age range for units 1 and 2 is between ~21,700 and ~16,700 cal yr B.P. (*25*).

##### Gault AUP lithic technology

The Gault Assemblage contained within units 1 and 2 at the Gault site is by far the largest collection of AUP artifacts in North America. Of these artifacts, 464 diagnostic and/or classifiable whole or fragmentary lithic artifacts were identified, including bifaces, projectile points, prismatic blade cores and blades, unifaces, gravers, burins, and modified flakes. Tools were made almost exclusively on local Edwards Plateau chert, which is available within the site at exposures along Buttermilk Creek.

**Fig. 4.**
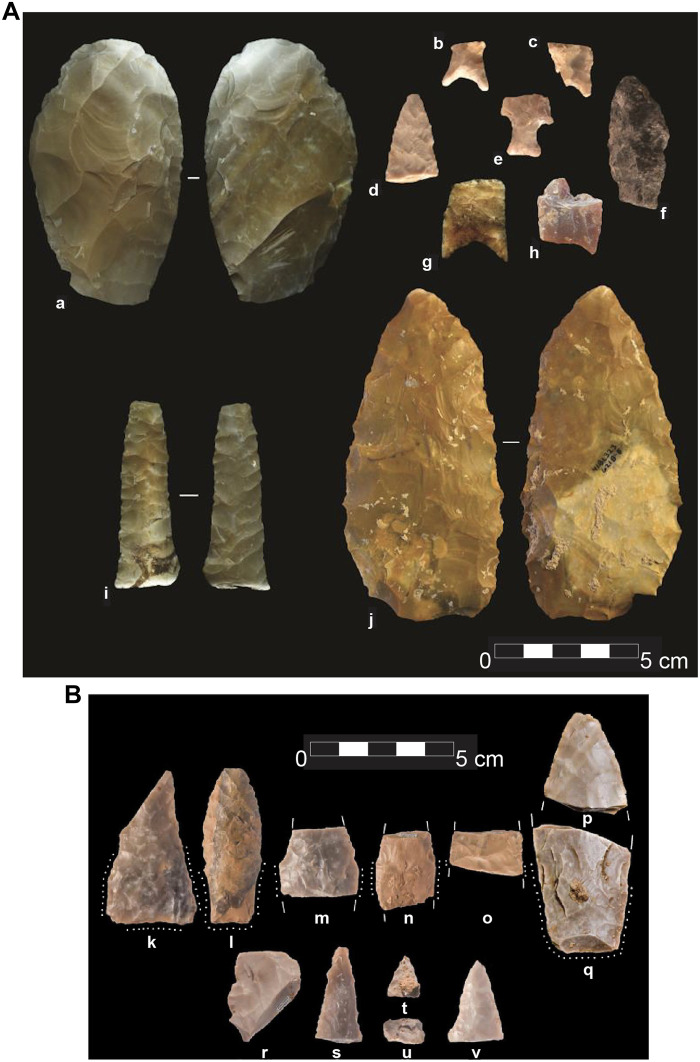
AUP bifacial tools and projectile points from the Gault and Debra L. Friedkin sites. (**A**) Examples of the biface technology in the AUP Gault Assemblage including large bifaces [(a) and (j)], projectile point fragments [(b) to (f)], and a lanceolate biface exhibiting collateral parallel oblique flaking (i). Modified from Williams *et al.* (*25*) [RightsLink license no. 609052136540]. (**B**) AUP projectile points from the Debra L. Friedkin site; (k) triangular lanceolate point, (l) lanceolate stemmed point, (m) lanceolate stemmed point midsection with base and blade sections, (n) lanceolate stemmed point midsection with base and blade sections, (o) lanceolate stemmed point midsection with base and blade sections, (p) point tip, (q) lanceolate stemmed point base, (r) point midsection, (s) beveled point tip, (t) beveled point tip, (u) beveled point tip midsection, and (v) point tip. Modified from Waters *et al.* (*30*) (RightsLink license no. 6090530177149).

The biface collection includes 38 whole or fragmentary bifaces and 10 projectile points, which are characterized by a focus on creating proportional cutting tools (Fig. 4A). Most of these were produced using collateral flaking for shaping and retouch. There are rare overshot and end thinning flakes, but their presence appears to be based on chance rather than design and the paucity of such flakes distinguish the Gault Assemblage from the overlying CPT assemblage (*25*). The bifacially worked projectile points include small stemmed and lanceolate forms (*25*). The only whole specimen is unusual in that it is produced from smoky quartz and is rather crude as a result. Of the remaining nine, five are basal fragments and four are undiagnostic tip fragments. Of the basal projectile point fragments, three are stemmed points with shoulders [Fig. 4A, (b) to (d) and (f)] and two other point fragments lack shoulders [Fig. 4A, (g) and (h)]. All but one of the stemmed points have slightly to moderately concave bases, and all the stems expand slightly from the neck to the base. All these stem fragments are small, with neck widths of 8 to 12 mm, basal widths of 12 to 16 mm, and haft lengths of 12 to 15 mm. Three of the points bear edge-ground haft margins [Fig. 4A, (b), (c), and (e)]. The single stemmed point, which retains a portion of the blade [Fig. 4A (f)], has beveling along one edge. The two fragments that lack shoulders have hafts that range from 21 to 22 mm at their upper limits to 19 to 22 mm at their bases and both have slight basal concavities. Only one of these unshouldered points is edge ground along its stem margins. The unshouldered point fragments are too generalized to be distinguished from later Paleoindian forms, but the other stemmed points are quite distinctive and are unlike any later Paleoindian or Archaic point forms found in the Texas region (*25*).

A distinctive core-and-blade industry is represented by 86 blades and blade fragments and six blade cores or core fragments. Three cores are complete enough to be characterized as either formal flat-backed blade cores (two) or semiconical cores (one). All three exhibit a similar manufacturing method used to produce a regular series of blades, including the establishment of a core platform, a unidirectional blade face, a flattened back, and modified sides (*26*). The production sequence maintained blade face curvature and allowed for the continued manufacturing of blades as is evident on longitudinally curved blade faces on the three cores. Most of the blades can be characterized as true blades [sensu (*27*)], but both crested and cortical blades are also present. The length of the complete blades ranges from 70 to 147 mm. Four of the blades have been retouched along one or both margins.

The Gault Assemblage shares broad similarities with the later CPT assemblage at Gault in terms of biface technology, blade production, and flake reduction (*25*, *26*). However, there are specific differences in all these areas, suggesting that as the Gault Assemblage technology was replaced by the patterns of the CPT, some aspects of technological toolkit were retained and modified, while others were substantially changed (*26*). While biface production constitutes a major component of the overall lithic technology in each assemblage, both the manufacturing strategies and the biface forms that were produced differ markedly. Biface manufacturing in the Gault Assemblage was used as a method adopted to produce nonprojectile point tools, whereas in Clovis, biface manufacturing appears to be specifically related to the production of Clovis points, with occasional use as expedient tools. Likewise, while blade manufacturing was also a major goal of lithic production in both assemblages, the types of cores and resulting blades in the two assemblages differ, suggesting that the Gault Assemblage blade manufacturing technology was “ancestral” to the more standardized CPT blade technology (*26*). The early lithic assemblage at the Friedkin locality, which dates to between the Gault and Clovis assemblages, is characterized by a more elaborated bifacial technologies and may be transitional between the two.

#### Debra L. Friedkin

The Debra L. Friedkin site, located ~500 meters downstream from Gault (Fig. 1), also preserves AUP components stratified below Clovis. Excavations at Friedkin uncovered a pre-Clovis archeological component known as the Buttermilk Creek Complex (*28*, *29*). During the initial fieldwork phase (2006 to 2009), 15,573 artifacts were recovered from a 20-cm-thick deposit beneath the Clovis horizon, including 83 tools and 15,490 pieces of debitage. This assemblage includes evidence of biface manufacture, blade and bladelet production, and core reduction strategies. A second excavation phase conducted in 2015 to 2016 expanded the assemblage to approximately 100,000 artifacts, including 328 tools and 12 complete and fragmentary lanceolate and stemmed projectile points (Fig. 4B) found 15 to 20 cm below the Clovis/Folsom zone (*30*). Among the tools confirmed from the ~15,500- to 13,500-year-old sediments are 40 late-stage biface fragments, as well as blade segments, bladelets, scrapers, a discoidal core, and snap fracture tools (radial and bend-break types), most commonly made on flakes but also on bifaces and retouched flakes. Artifact analysis is ongoing, and a full description of the 328 tools in the Buttermilk Creek Complex is forthcoming.

#### Cooper’s Ferry/Nipéhe

The Cooper’s Ferry/Nipéhe site is located along the lower Salmon River in west-central Idaho (Fig. 1), e.g., (*31*) (see Supplementary Materials). Geoarcheological study of two excavation areas at the site defined a stratified sequence of deposits, including a lower loess deposit lithostratigraphic unit 3/lithostratigraphic unit B3 (LU3/LUB3) that contains an AUP occupation with age controls provided by 18 ^14^C age estimates. Bayesian modeling of radiocarbon data places the start of occupation to between ~16,045 and ~15,725 cal yr B.P. Lithic debitage and stemmed projectile points recovered in situ outside and stratigraphically below dated pit features indicate an older as yet undated human occupation.

##### Cooper’s Ferry/Nipéhe AUP lithic technology

**Fig. 5.**
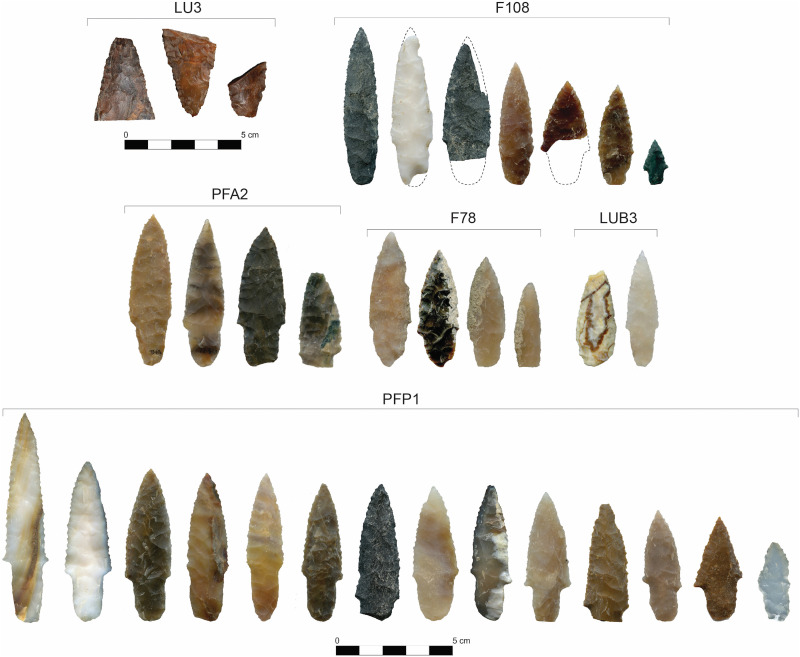
Stemmed AUP projectile points from the Cooper’s Ferry/Nipéhe site. Labeled brackets at top of each set of projectile points signify the features and lithostratigraphic units associated with these artifacts. Modified from Davis *et al.* (*31*) (RightsLink license no. 6090520467321) and Davis (*36*) [published under the Creative Commons Attribution license (CC BY 4.0, https://creativecommons.org/licenses/by/4.0/)].

Excavations of items deposited within the sediments of LU3 in Area A produced 161 pieces of debitage and 29 stone tools, including two Levallois-like cores, two fragmentary stemmed projectile points, a projectile point blade, four biface fragments, three prismatic macroblades, a burin spall, and 19 modified flakes (Fig. 5) (*31*). A pit feature, PFA2, was excavated downward from the surface of LU3 at ~13,210 cal yr B.P. and contained 724 pieces of debitage and 13 lithic tools, including four stemmed projectile points, two unifaces, three blades, two Levallois-like linear macroflake products, one unidirectional core, two modified flake tools, and a hammerstone (*32*–*34*). Three pit features in Area B, F78, F108, and F151, were constructed and buried in LUB3 sediments ~15,785 cal yr B.P. and contained a total of 12 complete and fragmentary stemmed projectile points, a biface fragment, a burin spall, a small fragment of a unidirectional core, and 321 pieces of debitage. A fourth pit feature (PFP1) was found within LUB3 sediments, but its upper limits were truncated by previous excavations conducted in the 1960s, thus obscuring its upper stratigraphic characteristics. This pit feature contained 14 stemmed points but no datable organic samples (*35*). Pedogenic calcium carbonate coatings were observed on the lower faces of each point, identical to those seen on other early points from F78 and F108 (*36*), which indicates that the PFA2 points were buried in LUB3 before the Rock Creek soil developed at the site ~14,000 cal yr B.P. (*31*, *36*). Two other stemmed points were found in LUB3 loess above and below the pit features.

#### Complementary/supporting American Upper Paleolithic assemblages

There are several other North American sites with lithic assemblages of the appropriate age, which have technological characteristics similar to those summarized here. Several of these have rather large stone tool assemblages, but issues with chronology and stratigraphic placement, make these assemblages difficult to assess. We consider only four complementary sites where we feel that chronological and stratigraphic controls are sufficient to allow comparison to the larger assemblages described above.

#### Page-Ladson

The Page-Ladson site is contained in a sinkhole along the Aucilla River in the Florida panhandle (Fig. 1), e.g., (*37*) (see Supplementary Materials). Comprehensive dating of the depositional sequence produced 71 ^14^C age estimates on wood preserved in the submerged deposits, with 24 of these coming from a stratigraphic column bracketing lithic materials. Seven, with an average age of ~14,550 cal yr B.P., are directly related to a biface and other flake tools (*37*). The site’s AUP lithic assemblage recovered during the two excavation phases is limited to a broken biface, two flakes showing evidence of use, and an additional 11 pieces of debitage (*37*, *38*). The flakes are described as bifacial thinning flakes, and the pointed biface is interpreted as functioning as a knife. One of the bifacial thinning flakes has a ground platform and is more than twice as long as it is wide. This blade flake may be derived from the initial forming of a blade core but by itself is insufficient to demonstrate that a core-and-blade technology was present at the site. Together, the pieces of debitage suggest that they were produced from the resharpening of at least three different tools (*38*).

#### Schaefer and Hebior mammoths

Schaefer and the nearby Hebior site are two mammoth (*Mammuthus primigenius*) butchering sites in southeastern Wisconsin located on the margin of what was the fluctuating Laurentide Ice Sheet at the time the bones were deposited (Fig. 1), e.g., (*39*, *40*) (see Supplementary Materials). The age of the Schaefer mammoth, which appears to have been butchered (*41*), is controlled by 13 ^14^C dates directly on mammoth bone and another 17 on wood macrofossils recovered from the peat in and around the bones (*40*), which average about 14,750 cal yr B.P.

While the lithic assemblage at Schaefer is limited to the two recovered blade flakes, they are diagnostic in that they represent the production of blade flakes for use as a principal tool type by AUP foragers. Both flakes are made from local chert. One of them is a prismatic blade, termed a “crest blade” by Joyce (*40*). This lame à crête would probably indicate use of a blade core technique to produce these blades. The Hebior mammoth was associated with two chert bifaces, a chert flake, and a flaked chopper made on dolomite (*39*, *42*, *43*).

#### Paisley Caves

The Paisley Caves are a set of small closely spaced caves overlooking Summer Lake in southeastern Oregon (Fig. 1), e.g., (*44*). Chronology is controlled by more than 400 ^14^C age estimates with more than 100 dating to earlier than ~12,800 cal yr B.P. Human fecal remains and stemmed points at the site date to before ~14,000 cal yr B.P., with the five earliest human coprolites dated to between ~12,500 and ~12,000 B.P. (~14,500 to ~14,000 cal yr B.P.) (*45*). Biface production was apparently the focus of the AUP knappers at Paisley Caves, although the number of formal tools is limited. Cutting tools consist only of a single biface and an edge-modified flake. The two older projectile points are broken but are both stem forms. The obsidian points were produced using bifacial collateral flaking, resulting in biconvex cross sections.

The stems on the two points are moderately long and tapered, ranging from ~25 to 40 mm from the base to slight shoulders where the blades begin. The distal ends of the stems are ~17 to 22 mm wide, with the stems tapering to slightly rounded bases of ~5 to 10 mm. The stem margins on the oldest point dating to between ~13,170 and ~13,314 cal yr B.P. are finely retouched and edge ground. The stem of the other possible AUP point is unground and has not been finely shaped by retouch. It may either have been broken during initial manufacture or was reworked and repurposed into a knife after it was broken. The shape and length of the blades are unknown.

There is no evidence of blade production in the Paisley Caves AUP lithic assemblage, but the number of tools in the collection is quite small, so the absence of blades may not be definitive. On the other hand, the amount of debitage is relatively large, and if blade production was common, then blade fragments might be expected to show up in the assemblage. Moreover, as Jenkins *et al.* (*44*) note, blades are extremely rare in later Western Stemmed Tradition (WST) assemblages, a Late Pleistocene to early Holocene cultural pattern defined by nonfluted stemmed and lanceolate projectile points found at many sites in western North America, e.g., (*46*–*49*). This WST pattern suggests that blades and formalized blade production may also have been absent at Paisley Caves.

#### Characterization of AUP technological attributes and patterns

A basic characteristic of AUP lithic technology is the production of linear flakes and blades from unidirectional, discoidal, polyhedral, and Levallois-like cores (Table 1). These flakes, blades, and spalls are used as informal tools and are further reduced to create a range of formal tools including bifaces, projectile points, gravers, unifaces, and burins. In some, but not all, AUP sites, projectile point production appears to be based, in part, on the relatively minimal reduction of flakes and blades, which often result in forms that tend toward plano-convex cross sections. Larger AUP biface technology is seen as well and appears to be based on more extensive reduction of larger flake spalls into bifacial preforms and finished bifaces with biconvex cross sections. AUP points are unfluted and appear in stemmed and lanceolate forms. AUP points are generally small, and examples from the Gault, Friedkin, and Cooper’s Ferry/Nipéhe sites are particularly diminutive—the smallest of which measured ~2 to 4 cm in length. AUP points appear in a range of two-dimensional hafting forms, including contracting rounded, pointed, and squared bases (Cooper’s Ferry/Nipéhe), concave and slightly contracting margins with squared bases (Meadowcroft), and expanding and slightly convex margins with square and concave bases (Gault). The stemmed AUP points generally have basal edge grinding, even including the smallest point form seen at the Cooper’s Ferry/Nipéhe site, which suggests that it was deployed in some kind of haft. Bifacial reduction is evident in the tools and debitage found at Cactus Hill, Meadowcroft, Gault, Friedkin, Cooper’s Ferry/Nipéhe, Hebior, Page-Ladson, and Paisley Caves. While bifacial collateral flaking to a midline is most common, overshot flakes were found in pre-Clovis contexts at Gault, Friedkin, and Cooper’s Ferry/Nipéhe. Further research is needed to determine whether this technological feature is unique to the CPT or has its origins in the AUP. The presence of smaller projectile points made on flakes or blades and larger bifaces made through more intensive reduction of bifacial preforms illustrates an important duality in AUP lithic technology that is reflected in later CPT and WST point forms where large and small point forms were used together. While the points were made through both bifacial reduction and modification of blade flakes, they can take the same general morphological shape. These shapes are varied in general, but small triangular forms are more common in eastern North America while stemmed forms are more common in the West. The AUP components at Cooper’s Ferry/Nipéhe also contain fragmentary prismatic blades made on cryptocrystalline silica and a unidirectional bladelet core made on fine grained volcanic material from PFA2 (*34*) and two fragments of prismatic blades made on chert.

#### Comparison of American and Northeast Asian Upper Paleolithic technologies

As Adovasio *et al.* (*23*) concluded decades ago: “Collectively, these data suggest that the first inhabitants of eastern North America employed a technologically standardized and sophisticated, small, polyhedral core– and blade-based industry of decidedly Eurasiatic, Upper Paleolithic ‘flavor’.” With the excavation and reporting of several additional well-dated AUP sites, this Upper Paleolithic “flavor” has become even more evident, leading to the obvious question of where this technology may have originated. The antecedents of the AUP may have been either in Europe or Asia, as Adovasio *et al.* noted, and a European origin for this American lithic technology has been suggested by some, e.g., (*50*). However, paleogenetic studies indicate that an Asian origin for Ancient Native American (ANA) populations (*51*) and the progenitors of AUP technology were most probably located somewhere in northeastern Asia. Unfortunately, these paleogenetic data are not yet sufficient to tell us where that location might have been.

One possibility is Beringia, the land bridge that connected Asia to the Americas during the Late Pleistocene. That bridge has long been held to be the most logical land route used by migrating ancient North Asians, e.g., (*52*), and until several North and South American sites were shown to predate the opening of an ice-free corridor between Beringia and the Americas south of the ice sheets, that route was long considered to be the only viable one. The hypothesis was so embedded in peopling of the America models that most of the same paleogenetic studies, which pinpointed a Northeast Asian source for those early populations, also identified an isolated standstill during that migration as a “Beringian standstill” without a shred of evidence that it took place in Beringia, e.g., (*53*, *54*). There are, however, good reasons to question that widely held assumption.

First, there are no known stone tool complexes in Beringia that are old enough and/or similar enough to be possible progenitors of AUP technology. All lithic tool assemblages in eastern Beringia postdate ~14,500 cal yr B.P., lack large core-and-blade production, and are characterized by a microblade technology and a small “teardrop-shaped” projectile point pattern that is not found in the AUP (*17*, *18*, *55*). Later lanceolate and stemmed point forms appear to have been introduced to eastern Beringia from lower-latitude North America (*56*). Second, what is known of Beringian paleogenetics suggests that Late Pleistocene populations there dating to ~11,500 cal yr B.P. did not contribute to the populations that first occupied North America south of the ice sheets (*57*). Similarly, paleogenetics suggest that pre–Last Glacial Maximum (LGM) populations in far northeastern Siberia did not contribute to ANA populations and that those early populations were replaced later by different groups from East Asia (*58*), in keeping with archeological evidence that northern Siberia was largely abandoned by humans during the LGM (*17*, *59*). In other words, present speculation that ANA populations and AUP technology originated in Beringia, e.g., (*7*, *8*), is just that—speculation—and so we need to look elsewhere for a likely cultural progenitor of the AUP. The archeological record of the northwestern Pacific Rim’s Paleo-Sakhalin-Hokkaido-Kuril (PSHK) peninsula and its vicinities in continental East Asia is older than the AUP, is geographically most proximal to North America, and is therefore the more likely region where an AUP cultural progenitor may be found. That too is largely speculation, given that there is much we still do not know about the Paleolithic technologies around the PSHK region, with part of that problem likely due to limited English language publications. Intensive study of collections will be needed to support a meaningful comparison between LUP lithic assemblages in both northeastern Siberia and the PSHK regions and AUP lithic technologies.

**Fig. 6.**
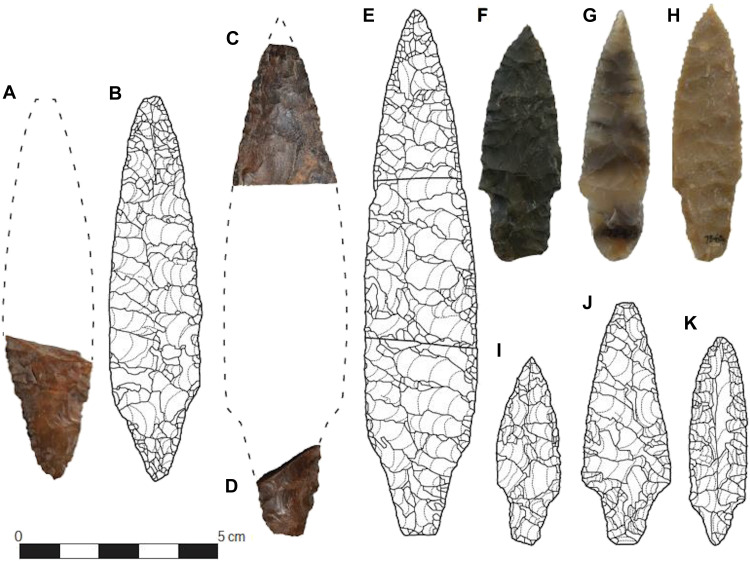
Comparison of stemmed points from Japan and North America. AUP points from Cooper’s Ferry/Nipéhe LU3 (**A**, **C**, **D**, and **F** to **H**) and bifacial stemmed points from Hokkaido (**B**, **E**, and **I** to **K**). Modified from Davis *et al.* (*31*) (RightsLink license no. 6090520467321).

**Fig. 7.**
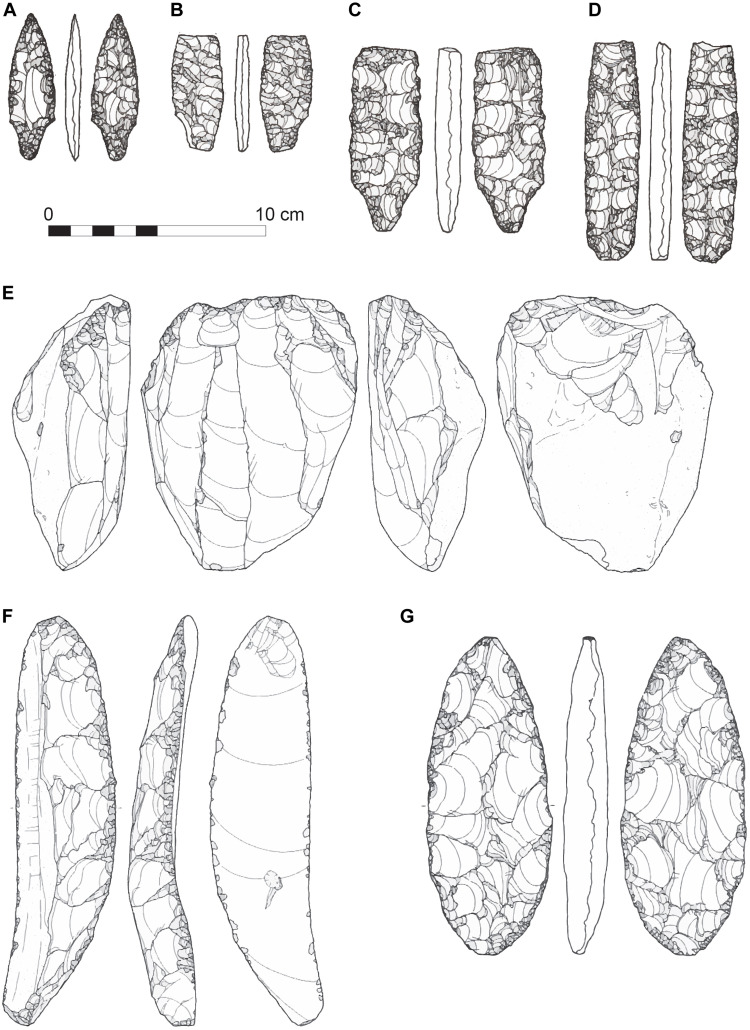
LUP artifacts from the Okushirataki 1 site, Hokkaido, Japan. Illustrated examples of bifacial stemmed points (**A** to **D**), blade core (**E**), blade tool (**F**), and biface (**G**).

Davis *et al.* (*31*, *36*), Davis and Madsen (*5*), and Buvit *et al.* (*60*) suggest that the pre-Clovis–aged large and small stemmed projectile point, core-and-blade, and biface technology found at AUP sites such as in the LU3/LUB3 deposits at Cooper’s Ferry/Nipéhe share design and manufacturing links with Late Pleistocene–aged lithic technological traditions observed in the PSHK region (Figs. 1, 6, and 7). Paleogenetic studies indicate that the progenitors of the First Americans share genetic ancestry with LUP peoples from both southern Siberia and far eastern Asia, becoming geographically isolated sometime after ~25,000 cal yr B.P. (*57*, *61*, *62*) before expanding into the Americas after ~19,500 cal yr B.P. (*62*, *63*). While paleogenetics has not yet pinpointed the exact location of their northeastern Asian residence, archeological evidence—particularly lithic technology—provides critical clues. The closest comparable projectile point forms in Northeast Asia to those predating ~16,000 cal yr B.P. found at the AUP sites discussed here are associated with LUP bifacial point-bearing sites in Hokkaido, (Fig. 6), e.g., (*31*, *36*, *60*).

#### Okushirataki 1

The Okushirataki 1 site was excavated during rescue campaigns conducted by the Hokkaido Archaeological Operations Center in 1997 and 2000. This site is important, as it provides a basis for establishing the age of LUP bifacial stemmed points in the PSHK region. A large excavation block covering 7752 m^2^ revealed 53 lithic scatters and 19 dense charcoal concentrations, primarily within an eolian sediment deposit designated as lithological unit IIa. In total, 830,243 lithic artifacts were unearthed, with 99,249 items found and mapped in situ. The site contains evidence of repeated occupation, beginning with an Early Upper Paleolithic (EUP) component characterized by a small flake-based tool assemblage. This was later followed by microblade technologies and bifacial points associated with subsequent LUP occupations (*64*–*66*).

Lithic scatter no. 53 at Okushirataki 1 yielded key evidence regarding bifacial stemmed points. A total of 9915 stone artifacts was recovered. Associated charcoal from feature no. 19 provided three radiocarbon dates with mean calibrated ages of ~21,400, ~21,360, and ~19,830 cal yr B.P. The youngest of these, ~19,830 cal yr B.P., is interpreted as the approximate age of the lithic assemblage. The elimination of older dates, combined with the occurrence of similar dates at other locations within the site, may suggest a past wildfire event. The lithic assemblage from this scatter includes bifacial projectile points, four of which being stemmed forms, as well as bifacial blanks or preforms, burins, end scrapers, side scrapers, and boat-shaped tools (Fig. 7). Other artifacts found at the site include retouched blades and flakes, prismatic blades, elongated flakes, blade cores, other cores, spalls, and a large quantity of debitage. Additional items such as anvil stones and a single obsidian nodule were also recovered. Obsidian dominates the lithic raw material assemblage, with minor occurrences of other cryptocrystalline rocks such as siliceous shale and agate. Chronological analysis of flake scar patterns, along with an extensive refitting analysis, indicates that the lithic artifacts in scatter no. 53 were primarily produced through blade-based, flake-based, and bifacial reduction techniques. The bifacial stemmed points, as shown in Fig. 7, were manufactured from the reduction of larger elongated flake blanks.

#### Hattoridai 2

The Hattoridai 2 site was excavated during 3 years of excavations conducted by the Hokkaido Center for Buried Cultural Property between 1998 and 2000 (*64*). The excavation covered a vast area of 6691 m^2^ on a very flat terrace along the Yubetsu River. A total of 65 lithic scatters and nine dense charcoal concentrations were found, primarily within eolian sediments of lithological unit IIa. In total, 798,648 lithic artifacts were unearthed, with 67,754 mapped in situ. The site contains an archeological sequence that begins with an EUP component, followed by an LUP component, which is characterized by various types of microblade technologies, and bifacial projectile points.

Lithic scatter no. 39 at Hattoridai 2 contains 3721 stone artifacts. Among these, 22 are bifacial projectile points, including 6 stemmed examples, along with 44 bifacial blanks or preforms, 3 end scrapers, 5 side scrapers, 6 retouched blades and flakes, 27 blades, 7 elongated flakes, 13 cores, 3 spalls, and 3594 flakes. Obsidian is the dominant raw material, with smaller amounts of siliceous shale, glassy andesite, and jasper. The artifacts were primarily produced through blade-based, flake-based, and bifacial reduction techniques. Charcoal from feature no. 5, which was a combustion feature directly associated with this lithic concentration, yielded three radiocarbon dates, with mean calibrated ages of ~16,750, ~16,620, and ~16,170 cal yr B.P. (*64*).

For the purpose of comparing AUP and eastern Beringian lithic technologies, the artifact assemblages from lithic scatter 53 at Okushirataki 1 and lithic scatter 39 at Hattoridai 2 lack microblade and microcore technologies, distinguishing them from other LUP toolkits in the region. However, the relative chronology and uses of microblades versus bifacial stemmed points in Hokkaido are complex and have been major research topics among local archeologists for a long time. Several assemblages contain both microblade reduction technology and bifacial stemmed points, although not always. For example, Hirosato- and Oshorokko-type microblade cores are found alongside Tachikawa-type bifacial stemmed points at sites such as Yoshizawa, Motomachi-2, and Nakamoto, while other bifacial point assemblages, such as those at Ochiai and Satsunai N sites, lack microblade reduction (*67*, *68*). The reasons for this variation remain unclear. Some researchers attribute it to temporal changes, while others suggest functional and contemporaneous differences between sites, but both interpretations lack solid evidence.

Microblade technology appeared in PSHK around the onset of the LGM and began to spread in southern Siberia ~23,000 cal yr B.P. (*59*, *60*, *69*). It continued to spread across Siberia, particularly in regions where these novel inset weapons served, in part, as a solution to the challenges of provisioning during long, harsh winters—supporting tactics adapted to the risks of cold-weather large-game hunting (*70*). Nonetheless, microblade technology is also found in later periods, for example, as far south as southern Kyushu, Japan, in the LUP context (*71*). It has even been controversially argued to be in association with pottery by 15,000 cal yr B.P. (*72*). Therefore, the appearance, design, and functional purposes of microblade technology in relation to environmental conditions may require further assessments. In eastern Beringia, microblade technology first appears after 14,500 cal yr B.P., followed by the emergence of smaller, thinner, teardrop-shaped bifacial projectile points shortly after 14,000 cal yr B.P. (*17*). Microblade technology, despite its utility in Siberia and Beringia, was not transmitted into AUP sites south of eastern Beringia during the Late Pleistocene for reasons yet to be fully explained.

**Fig. 8.**
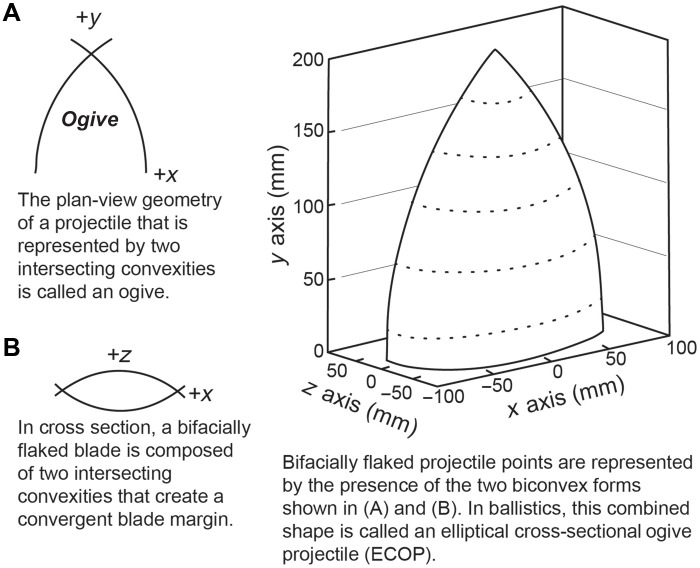
ECOP definition. Definition of bifacially flaked projectile points as elliptical cross-sectional ogive projectile (ECOP) based on the plan-view geometry (**A**) and the cross-sectional form (**B**) of its blade element.

The appearance of fully bifacially flaked, stemmed projectile points in the PSHK region after ~20,000 cal yr B.P. marks a critical innovation in ranged weapon systems. This innovation introduced the bifacially flaked elliptical cross-sectional ogive projectile (ECOP) design (Fig. 8), characterized by blade margins that converge to a pointed tip, forming a two-dimensional ogive shape with intersecting convex curves. While the ogive form is an outline shape that is commonly used in modern cylindrical bullets and missile nose cones, bifacial projectile points differ in their flattened elliptical bifacial cross section, which enhances strength and durability (*73*) and resharpening potential. Earlier linear blade points, lacking an elliptical bifacial cross section, were likely less robust and less as effective as a durable, maintainable ballistic weapon as these later bifacially flaked ECOP designs. The design and manufacturing processes of bifacial projectile points, therefore, are critical to the argument for a cultural connection between the AUP and the LUP of the PSHK region.

Before ~19,830 cal yr B.P., extensively bifacially flaked, stemmed projectile points were absent in Asia. Instead, sites in the mainland dating to this period demonstrate the use of composite microblade projectile technology, e.g., (*74*–*76*), as well as projectile points made on linear blades with minor pressure flaking to sharpen the tips and create hafting elements (*74*). The absence of bifacially flaked ECOP technology in mainland Asia before ~19,830 cal yr B.P. and in Beringia before ~14,000 cal yr B.P. suggests that this region was not the source of AUP projectile point technologies. Instead, the ECOP forms originating in the PSHK region provide the most direct technological link between Late Pleistocene Northeast Asia and North America. While Late Pleistocene populations in the PSHK region may have been familiar with both microblade and bifacial projectile point technologies around the LGM, if they were the source population, only bifacial ECOPs, prismatic blades, and bifacial tools were brought into North America south of the continental ice sheets after ~20,000 cal yr B.P.

Potter *et al.* (*8*) argue that stemmed projectile points cannot be used as a diagnostic cultural marker to link Late Pleistocene populations in North America and the PSHK region, stating that “stemming is a widespread form of haft design innovated numerous times across multiple continents and is thus not an appropriate derived character on which to base a hypothesis of cultural affiliation.” However, in focusing on haft design, Potter *et al.* (*8*) seem to be missing the point. While we agree that relying solely on haft design as a taxonomic attribute is problematic, their critique overlooks the broader significance of bifacially flaked ECOP blade technology as a diagnostic technological characteristic and transformative innovation. The invention and transmission of ECOPs fundamentally reshaped projectile weapon systems and likely played a critical role in enabling human migration into the Americas during the Late Pleistocene. One explanation for the continued adoption of the ECOP design in the Americas is that the development of ECOP technology during the LGM in PSHK may have been a key departure from earlier lithic traditions, such as microblade-based composite projectile systems that dominated human migrations to Siberia and later into Beringia. Unlike earlier technologies in PSHK and Siberia, which relied on composite systems of microblades combined with organic components, ECOPs introduced a bifacial blade form with an elliptical cross section. We hypothesize that this innovation enhanced strength, durability, and the ability to resharpen, improving overall weapon performance and marking a fundamental shift in lithic manufacturing strategies. Crucially, it was ECOP technology—not microblade technology—that was carried into North America by populations moving south of the continental ice sheets. The appearance of fluted points—another form of ECOP—in eastern Beringia postdates the emergence of the CPT in mid-latitude North America and seems to have spread northward through the ice-free corridor after ~12,700 cal yr B.P. (*77*, *78*). Although the reasons for the delayed decision in adopting the ECOP design weapons by foragers in eastern Beringia requires investigation, evidence underscores the fact that Late Pleistocene ECOPs did not originate in Beringia. ECOPs are present in pre-Clovis AUP sites south of the ice sheets well before their appearance in Beringia, underscoring their foundational role in the technological toolkit of the earliest populations in the Americas. As a key innovation, ECOPs provide an evolutionary link connecting Paleoindian developments, earlier AUP practitioners, and their origins in PSHK. Emerging during the LGM in PSHK, ECOPs mark an important advancement in lithic technology, bridging the technological lineage from the LUP of PSHK to the AUP and, ultimately, to the Paleoindians. By focusing on ECOP technology rather than the various forms of stemmed hafting, we can identify a meaningful connection between PSHK and AUP populations. The temporal and geographic pattern is clear: The ECOP design originated in or near PSHK during the LGM and spread southward into mid-latitude North America with early human migrants, before appearing in Beringia, and ultimately served as the cornerstone for the development of later Paleoindian lithic traditions.

While these technological similarities suggest a possible cultural connection between LUP manifestations in the PSHK and the AUP, the nature of paleogenetic connections are largely unknown. There is some evidence that Jomon populations did not contribute to AUP populations (*79*, *80*), suggesting a non-Japan origin. Jomon cultural manifestations (e.g., pottery) appeared in the Japanese archipelago south of Hokkaido as late as ~15,500 cal yr B.P. (*72*, *81*, *82*) but possibly as early as ~17,000 cal yr B.P. (*83*). The Jomon cultural pattern is recognized on Hokkaido at only two sites. One is the Taisho 3 site, where dates on charcoal residue on pottery range from ~14,700 to ~13,900 cal yr B.P. (the actual age of these sherds may be somewhat later due to an unknown marine reservoir effect) (*68*, *72*, *84*). The other is the Tachikarushunai M-I site, dating to ~15,100 to ~13,800 cal yr B.P., e.g., (*83*). In addition, technical analyses of the lithic assemblages from non–pottery-bearing Incipient Jomon sites in Hokkaido (e.g., at the Kyushirataki 5 site) show that they are dominated by willow-leaf projectile points with denticulate edges (*83*). These bifacial points, and associated pressure flaking patterns, are distinct from contemporary Terminal Upper Paleolithic sites (*85*). Because the flaking patterns of the Hokkaido Jomon lithic assemblage are similar to the Jomon sites to the south, Natsuki (*83*) suggests that the Incipient Jomon left Hokkaido and moved to Honshu during cold conditions coinciding with the Younger Dryas (~13,000 to 11,500 cal yr B.P.). Later, the Initial Jomon reimmigrated to Hokkaido, where their society developed from a blend of the LUP traditions of Hokkaido and the Jomon culture of Honshu.

Recent genomic advances suggest that the Jomon people were direct descendants of LUP groups that migrated into the Japanese archipelago from Southeast Asia (*86*). Nonetheless, potential differences between the Jomon lineage and LUP populations of Hokkaido remain poorly understood. Notably, recent studies imply the LUP groups of PSHK were likely genetically distinct from the ancestral Jomon population of Honshu, e.g., (*87*), supporting the view that northern groups that developed microblade technology during the LUP were not descendants from the southern groups, e.g., (*86*). Given the AUP chronology discussed above, we suggest the parent LUP population in Northeast Asia dates to before ~20,000 cal yr B.P., and until human skeletal remains older than that age are found in the PSHK and nearby regions of Northeast Asia, it will remain impossible to use genetics alone to speculate on possible locations of antecedent AUP populations. However, genomic studies leave open the possibility of a “ghost population” existing in Hokkaido during the LUP. The distinctive technological features seen in Hokkaido LUP lithic assemblages dating to between ~20,000 and ~16,000 cal yr B.P. provide some of the strongest clues potentially linking the cultural patterns of the LUP in the PSHK to the AUP. While we cannot yet determine whether the LUP populations on Hokkaido or elsewhere in the PSHK (or Beringia, for that matter) were directly genetically related to the First Americans, their technological lithic traditions do suggest potential cultural connections. By focusing on these commonalities, it may be possible to more definitively identify whether the region served as a source of technological or cultural progenitors for the tools and technologies that later appeared in the Americas.

#### The end of the American Upper Paleolithic period

While the beginnings of the AUP remain unclear and will likely always remain so given the unlikelihood of finding the single earliest human occupation of the Americas, the end of the AUP is relatively well defined, both chronologically and in terms of the diagnostic tools, which mark the shift to the following Paleoindian period. About 13,000 cal yr B.P., across most of the Western Hemisphere, there was a marked shift to the use of large sturdy lanceolate points probably used to tip throwing or thrusting spears and javelins. Concurrently, the production and use of large core-and-blade implements ceased to be an important part of the lithic toolkit, although this shift was more rapid in some places than in others.

In western North America, Bayesian modeling of associated ^14^C age estimates suggests that the use of large lanceolate Haskett-type stemmed projectile points became widespread beginning sometime between ~13,260 and ~12,895 cal yr B.P. (*3*, *88*). These stemmed points, with ECOP blade designs and stemmed hafts that are typically longer than the blade portion of the projectile, reached up to 226 mm in length and weighed as much as 61.8 g (*89*). Relatively large and robust stemmed points occur in the AUP levels at Cooper’s Ferry/Nipéhe, reaching 67 mm in length (a broken point may have been up to 10 cm in length) and weighing as much as 9.24 g, but the advent of the considerably larger Haskett variety stemmed points represents an elaboration of this form. As Duke and Stueber (*89*) note, Haskett and Clovis points are similar in terms of size, function, technological production techniques, required knapping skills, and the ways in which they are reworked or resharpened. Haskett and Clovis, according to Duke and Stueber (*89*), “…share a system of repair, long use life, and multipurpose functional trajectory that has some quantifiable measure of similarity.” While prismatic blade and Levallois-like cores persist into the early Holocene at a few sites such as Cooper’s Ferry/Nipéhe, for the most part core-and-blade technologies, one of the primary characteristics of AUP assemblages, disappear concurrently with the appearance of Haskett points. The number of WST sites with large lithic assemblages directly dated to ~13,000 cal yr B.P. is limited, but there are thousands of WST sites around many of the terminal Late Pleistocene to early Holocene-aged lakes in the Great Basin with surface assemblages dating to ~13,000 to 8500 cal yr B.P.; all of which lack core-and-blade components, e.g., (*49*).

In central and southeastern North America, similar large, robust Clovis lanceolate points appear at about the same time. Bayesian modeling of ^14^C age estimates associated with Clovis points suggest that they first appear sometime between ~13,275 and ~12,980 cal yr B.P. (*3*). This age range begins slightly later than a similar chronological modeling effort, which includes sites without direct evidence of Clovis diagnostics (*2*). In that regard, there are actually fewer radiocarbon age estimates directly associated with Clovis assemblage diagnostics than are associated with AUP assemblages. Unlike the WST sites to the west, Clovis assemblages retain some core-and-blade tool production strategies, but these are elaborated to include the use of large conical cores to produce elongate blade forms (*90*, *91*). Even these blades forms were largely abandoned by the end of the very short ~600 to ~250-year Clovis period (*3*) when Clovis fluted point styles were elaborated into those found in later Folsom assemblages (*90*). What caused such a marked shift in projectile point forms and functions between the AUP and the following period of lanceolate point use is as yet unknown, although the nature and timing of that shift are clear. Because what is known about the (probable) varied nature of AUP subsistence and settlement systems is extremely limited, as indeed what is known about adaptations during the following lanceolate period, any hypotheses about what may have led to this technological shift would be largely speculative. We suspect that the shift may be related to the gradual extirpation of many Late Pleistocene mammal species during the Bølling-Allerød climatic period between ~14,700 and ~12,900 cal yr B.P. and to a marked reduction in the population of many other large-bodied species, such as mammoths, by the end of the following Younger Dryas, e.g., (*92*).

## DISCUSSION

Over the course of the last 40 years, the number of well-dated, stratified sites in North America with initial occupations dating to before ~13,500 cal yr B.P. has gradually increased to the point where there are now nearly as many AUP sites as well-dated Clovis sites. Initial attempts to characterize regional variation among these sites were based largely on identifying differences in projectile point morphologies, e.g., (*1*), due primarily to a lack of lithic assemblages large enough to characterize any fundamental technological similarities these AUP sites may have shared. The apparent differences in point morphologies remain, but there are as yet too few AUP sites to determine whether these represent real regional differences or merely functional differences in site use and hunting strategies. Despite these differences in point morphologies, there is now a large enough sample of reasonably well-dated AUP sites with relatively large artifact assemblages to begin to identify underlying technological similarities in stone tool production. In turn, it is beginning to be possible to search for archeological assemblages in the world, which have both similar tool production strategies and are old enough to have served as possible progenitors of the fundamental AUP lithic technology.

We have identified 10 sites located in North America below the limits of the LGM ice sheets, which together serve as the basis for understanding the nature of the AUP technologies. These include Cactus Hill in Virginia, Cooper’s Ferry/Nipéhe in Idaho, Gault and Friedkin in Texas, Meadowcroft in Pennsylvania, Page-Ladson in Florida, Paisley Caves in Oregon, Schaefer and Hebior in Wisconsin, and White Sands in New Mexico. Of these 10, the first 5 sites have AUP lithic assemblages large enough to adequately characterize their underlying tool production strategies, with the remaining 5 providing ancillary support. There are other possible AUP sites that may yet be included in this group, but questions about associated dating, relationships between age estimates and artifact assemblages, and whether or not the sites even represent human occupation make their interpretation problematic. Similarly, we restrict our analysis to North America because of the complexity of the South American record and the limited number of sites dating to the AUP period.

Except for the Schaefer, Hebior, and White Sands sites, these North American AUP sites are all stratified and contain post-AUP components with diagnostic artifacts associated with age estimates corresponding to similar diagnostics widely found elsewhere. Age estimates associated with the basal AUP components at these sites range between ~20,000 and ~13,500 cal yr B.P. Because the 10 sites are widely distributed across North America south of the ice sheet, the AUP populations associated with their occupations were also apparently widespread, albeit perhaps thinly. Given this wide distribution and the associated age range, we speculate that the initial occupation of lower-latitude North America likely occurred around 20,000 cal yr B.P.

Stone tool production at these sites is broadly similar, involving both core-and-blade and biface production techniques and the manufacture of ECOP point designs. In the former, flat-faced or Levallois-like cores were used to produce elongate blade flakes, which were used without further modification as simple cutting or slicing tools or further unifacially or bifacially modified into a variety of other tool forms. Many tools were also produced by bifacially and bilaterally reducing large cores or flakes into a variety of desired tool forms. Both the core-and-blade and bifacial techniques were used to support the production of a variety of lanceolate and stemmed projectile points. These are usually small, often less than 5 cm in length but occasionally reach up to ~10 cm long at sites such as Cooper’s Ferry/Nipéhe. The stemmed point forms have both expanding and contracting stems, with the stems comprising less than half the point form.

These dual LUP production techniques are common throughout Eurasia, as Adovasio *et al.* (*23*) noted long ago, and it is necessary to use a combination of specific technological characteristics and paleogenetics to more tightly pinpoint where the AUP may have originated. These dual production technologies are also present in many European Upper Paleolithic technologies (*93*) but connecting the two is more problematic (*94*). Paleogenetics suggest the founding North American AUP populations formed somewhere in Northeast Asia when North-Central Eurasian populations merged with East Asian populations sometime before ~20,000 cal yr B.P. (*51*). Exactly where in Northeast Asia this merger, which led to the origins of AUP people, took place cannot be determined by paleogenetics alone, but simple geography suggests that it is likely to have occurred somewhere in the PSHK area in what is now northeastern China and the southern Russian Far East, with a North-Central Eurasian population moving eastward, possibly along the Amur River, and an East Asian population moving northward along coastal East Asia (*5*).

It is then reasonable to ask where within this rather broad geographical area are there archeological complexes old enough and similar enough to AUP technologies that they may represent the locations where AUP populations originated. During the LGM before ~20,000 cal yr B.P., the PSHK consisted of broad peninsula extending from the mouth of the Amur River southeast toward the islands of what is now Japan; thence, running northeast along a string of very closely spaced islands (the Kurils) toward Kamchatka. What is now the island of Hokkaido formed the southern extension of this peninsula but was separated from paleo-Honshu, the main island of Japan, by a narrow strait. While archeological research in much of the PSHK is limited, a number of LUP sites on Hokkaido have been excavated, dated, and reported. Some LUP components at these sites contain examples of flat-faced cores, large prismatic blades, large bifaces, and large and small stemmed bifacial projectile point technologies that date as early as ~20,000 cal yr B.P. (*64*). They differ from the AUP in that many of these sites also contain microblades, which are absent in the AUP.

**Fig. 9.**
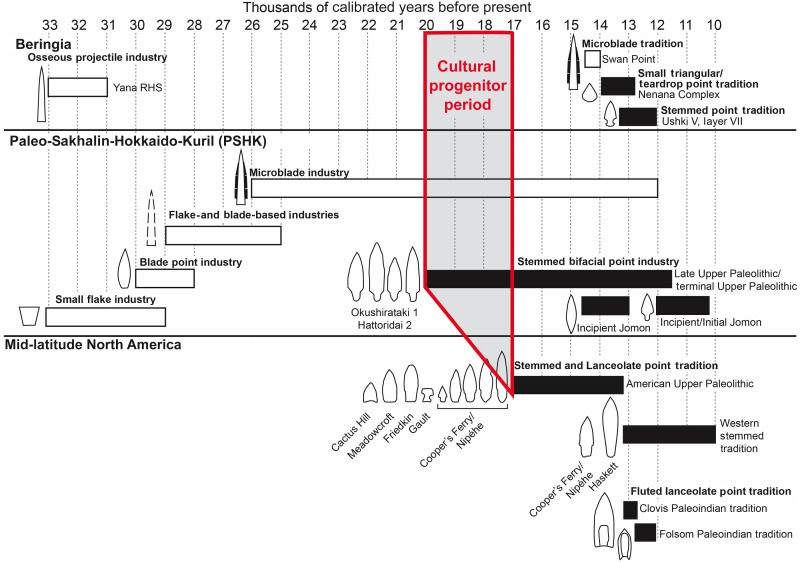
Summary diagram showing the chronological relationships between lithic assemblages in the PSHK, Beringia, and the AUP. Note the complete absence of lithic assemblages dating to between ~30,000 and ~15,000 cal BP in Beringia. Note also that the common features of AUP assemblages appear several thousand years earlier in PSHK stone tool assemblages. Finally, note the chronological relationship between the stemmed and lanceolate points of the AUP, and the appearance of the larger, and more robust lanceolate Haskett and Clovis points characteristic of the later Western Stemmed Tradition and Paleoindian periods.

Given the relative lack of archeological research in the remaining greater PSHK region, it is difficult to specify Hokkaido as the source of AUP populations and the technological complexes which accompanied them, and we do not do so here. These Hokkaido LUP assemblages are certainly of the right age and the right technological composition to have served as the progenitor of the AUP complexes and are also well within the greater Northeast Asian geographical area where proto-ANA populations formed and underwent a “stillstand” of several thousand years (*51*). It is also possible that similar archeological complexes may be found elsewhere in the PSHK or its margins. It seems unlikely, however, that this stillstand occurred north of the PSHK in Beringia, given the absence of archeological sites of the right age and the apparent abandonment of Beringia during the LGM (Fig. 9) (*17*, *60*). The absence of microblades in AUP lithic assemblages is intriguing, given their presence in lithic assemblages dating to well before ~20,000 cal yr B.P. on Hokkaido and elsewhere in Northeast Asia (*17*, *60*, *66*, *76*, *95*). There has been some suggestion that the appearance of microblades represents an adaptation to the colder northern latitude conditions of the LGM, e.g., (*70*, *96*, *97*); although they are found as south as the southern Kyushu region of Japan, e.g., (*71*, *98*) with undetermined functions. If proto-AUP populations migrated out of PSHK carrying both microblade and bifacial technologies, then we speculate that the microblade component—adapted to cold, harsh northern environments—became less necessary, as these populations moved into southern North America. Consequently, microblade technology was largely abandoned in favor of a fuller reliance on stemmed and lanceolate bifacial projectile points, along with whatever “soft” technologies were more effective in these new environments. Just such a pattern is found on Hokkaido, where the use of microblades largely disappeared during the terminal Pleistocene/Holocene transition, as the climate warmed and the environmental conditions associated with the end of the LGM became more favorable (*96*).

If the PSHK was the source for the founding population in the Americas, when, how, and by what route did this migration occur? Although the exact timing is uncertain, the ice-free corridor between the Laurentide and Cordilleran ice sheets appears to have closed by around 26,000 cal yr B.P. (*99*) and remained impassable until after the appearance of AUP sites south of the continental ice sheets (*100*). This suggests that the initial migrants into the Americas did not use an interior land route. Unless, that is, sites in the Americas dating to older than the age of this closure can be confirmed. Barring such confirmation, a Pacific coastal route seems to have been most likely (*60*, *100*). Two possible variants of this route have been proposed (*5*): a complete coastal route from the PSHK to southern North America via coastal Beringia and/or the Aleutian archipelago or a modified route from Beringia to the Alaskan coast and thence southward. Given the absence of sites in Beringia during the LGM, the former route is the more parsimonious. On the basis of the age of well-documented AUP sites in North America, we suggest that this initial migration occurred sometime about 20,000 years ago. Praetorius *et al.* (*101*), in a review of environmental conditions along the Pacific coast of North America, particularly variations in the strength of counterclockwise coastal currents, suggest that the most optimal times for a coastal migration during this period were between ~24,500 and ~22,000 cal yr B.P. and again between ~20,000 and ~19,000 cal yr B.P. Similarly, on the basis of an examination of faunal records from the same southern Alaska and Canadian coastal zone, Steffen (*102*) concludes that glacial ice cover “probably hindered” migration starting between ~23,300 and ~20,000 cal yr B.P. and lasted until ~18,900 to ~17,700 cal yr B.P. Together, these studies conclude that conditions were optimal for a coastal migration into the Americas sometime about 22,000 years ago, a time estimate compatible with our archeologically based estimate of sometime shortly before ~20,000 cal yr B.P.

Some of this speculation, however, is implicitly based on the notion that the initial movement of people into the Americas was based on long-ranging, rapid, sea voyages undertaken against strong oncoming currents. However, we really have no notion of how long such a circum-Pacific migration might have taken. This migration may have taken place over the course of a thousand years or more, lasting 40 to 50 generations and involving a series of movements of people from one place to another relatively nearby location. This wave of advance population movement may easily have used short coastal voyages in waters between off-shore currents and the wave breaker zone and could have enabled such migrants to access coastal refugia, which became differentially available through time (*5*, *102*). If so, then relatively adverse environmental conditions may not have been an obstacle, especially because migration may have been difficult during any period of the LGM. Alternatively, the migration may have been rapid but also have occurred any time. By ~30,000 cal yr B.P., Upper Paleolithic seafarers were using sea-going vessels to access some of the outer islands in the Japanese archipelago, e.g., (*103*) and were capable of negotiating the Kuroshio Current, one of the fastest in the world (*104*, *105*). This suggests that such experienced seafarers may also have been capable of handling adverse Pacific coastal currents. Either way, environmental constraints on the timing of the initial occupation of the Americas may not have been much of a factor.

### Major points

1) Paleogenetics suggest that the founding population in the Americas formed somewhere in northeastern Asia about 25,000 years ago and, after a ~5000 to 4000-year stillstand or bottleneck at some localized area within that region, expanded into the Americas south of the ice sheets sometime after ~20,000 cal yr B.P.

2) Because paleogenetics cannot yet tell us the specific location where this stillstand occurred, it is necessary to use archeological evidence to build arguments regarding where that specific location may have been.

3) On the basis of the dating of 10 sites located across southern North America, initial occupations occurred between ~18,000 and ~13,500 cal yr B.P., possibly as early as ~23,000 cal yr B.P. Because such a widespread distribution may have taken at least several thousand years to achieve, archeological evidence discussed here also suggests that an initial occupation likely occurred around 20,000 years ago or slightly before.

4) We suggest that early seafarers, well adapted to the use of boats on the open ocean, probably followed a circum-Pacific coastal route into the Americas from the PSHK sometime about 20,000 cal yr B.P. or slightly before. This migration was probably gradual, as members of this population moved from one refugium to another within generally hostile LGM northern Pacific environments.

5) While the lithic assemblages at these early sites show some variation in projectile point morphologies, they share an underlying technological similarity in the way stone tools were produced.

6) Similarities include the use of elongate blades made on a variety of core forms and the bifacial production of other tools, including stemmed projectile points.

7) This dual use of core-and-blade and bifacial reduction techniques is common to many LUP assemblages throughout Eurasia, but the production of bifacial points bearing an ECOP design is regionally uncommon or absent before the LGM.

8) Bifacially flaked ECOP-based stemmed points, as well as elongated blade production and large bifacial tools, are found in assemblages recovered from the southern PSHK region dating to around 20,000 cal yr B.P. These projectile points share specific design elements and production techniques with stemmed points found in the AUP.

9) During the LGM, when global eustatic sea levels were more than 100 m lower than at present, the PSHK was a peninsula extending from the mouth of the Amur River in the Russian Far East to the Japanese archipelago and northward along a string of closely spaced islands to Kamchatka on the southern margin of Beringia.

10) Given the absence of archeological sites in Beringia dating to the LGM, combined with the presence of antecedent lithic assemblages that predate but resemble the AUP, the isolated PSHK region emerges as a logical location for the formation of the population ancestral to the First Americans.

11) The precise location of the initial AUP population within the larger PSHK region or its margins remains unknown, but the northern Japanese island of Hokkaido is a strong candidate.

## Supplementary Materials

### This PDF file includes:

Supplementary Text Figs. S1 to S9 References
