# Supplementary material for: Characterizing the American Upper Paleolithic
Source: Sci Adv. 2025 Oct 22;11(43):eady9545. doi: 10.1126/sciadv.ady9545 (PMC12542946; doi:10.1126/sciadv.ady9545)
Supplement: Supplementary file 1 — Supplementary Text Figs. S1 to S9 References [file sciadv.ady9545_sm.pdf]

Science Advances
AAAS

Supplementary Materials for

Characterizing the American Upper Paleolithic

David B. Madsen *et al.*

Corresponding author: Loren G. Davis, loren.davis@oregonstate.edu

Sci. Adv. **11**, eady9545 (2025)
DOI: 10.1126/sciadv.ady9545

This PDF file includes:

Supplementary Text
Figs. S1 to S9
References

Site Descriptions

Here we provide brief descriptions of the setting, stratigraphy, and chronology of the 10 American Upper Paleolithic (AUP) sites discussed in the main text.

Cactus Hill

Cactus Hill is a stratified dune site adjacent the Nottoway River on the coastal plain of southern Virginia (Fig. S1) (*19, 106-108*). Laminated sand layers at the site contain early Archaic, Clovis, and AUP deposits separated by 10–20 cm of culturally sterile sand (*20*). Chronology at the site is controlled by both Optically Stimulated Luminescence (OSL) age estimates and several ^{14}C dates (*109*). The earliest cultural deposition is bracketed by a date on charcoal from a hearth associated with overlying Clovis materials of 10,930±250 BP (13,350-12,430 cal BP at 2σ) and by six dates on various organic remains from underlying strata of ~23,800-20,200 cal BP (*109*). A charcoal sample collected from 2 cm below where this conventional age came from is more precisely dated by AMS to 16,940 ± 50 BP (20,580-20,320 cal BP at 2σ). These dates, and the bracketing ages of ~20,200-~12,200 cal BP, are consistent with four calculated OSL age estimates from the earliest cultural layers that fall between ~23,000 and ~17,100 cal BP. These age estimates suggest the initial occupation of Cactus Hill was ~18,000 cal BP or earlier, but there are several dating issues which contribute to uncertainty about this very early age (e.g., *110*). Regardless, the relative stratigraphic separation between the AUP and CPT horizons and the differences in technological characteristics between the lithic materials in the two horizons make it clear the lowest cultural deposits date to some time prior to the CPT era. Four cultural features consisting of combinations of charcoal, flake/blade clusters and calcined bone fragments were discovered during excavation of the Pre-Clovis levels at Cactus Hill (*19*: Table 12.5). Two of these working surface/hearth features were directly dated. The first includes burned debitage and bone fragments, and blade fragments associated with a hearth feature that returned a radiocarbon age on wood charcoal of 14,180±80 BP (17,415-17,050 cal BP at 2σ). The second feature included burned blades and debitage associated with a hearth that contained charcoal dated to 15,070±70 BP (18,645-18,430 cal BP at 2σ). In addition, there were a number of individual blade clusters which were not formally identified as cultural features.

Meadowcroft Rockshelter

Meadowcroft is a well-stratified rockshelter along a tributary of the Ohio River in western Pennsylvania spanning the entire precontact period (Figs. S1 & S2). While a comprehensive monograph reporting the late 1970s excavations has yet to be published, more than 20 papers and articles on the site are available (see *23*, and references therein). More than 50 radiocarbon dates from the site are available for the 11 stratigraphic units. While they are in the correct stratigraphic order and are associated with diagnostic artifacts of the appropriate periods, the ages for the earliest AUP occupations have been questioned because of a suggestion that the dated charcoal fragments were contaminated by seepage from a nearby coal seam (e.g., *110, 111*). That hypothesis has been tested and rejected (*112*). The dates have also been questioned because "The recovered fauna and flora are discordant with other local Wisconsinan age fossils" (*111, 113:* 580). This too is a misplaced criticism since deciduous trees such as beech (*Fagus* sp.) and red maple (*Acer rubrum*) occupied refugia just south of the ice sheets (e.g., *114, 115*). However, the radiocarbon dating of lower Stratum IIa, the lowest cultural stratum at the site, remains problematic not because the dates are inaccurate but because they are conventional ^{14}C age estimates with very large standard deviations. As a result, the ages may be accurate, but are not very precise and in some cases could fall anywhere within a 4,000-year time span at 2σ. Standard dates on charcoal from six firepits in the lower 1/3 of Stratum IIa range in age from 10,850±870 to 14,255±975 BP (15,010-10,240 cal BP to 19,510-14,315 cal BP at 2σ) and Adovasio et al. (*21*: 335) suggest AUP people were definitely present at the site "…sometime between 13,955 and 14,555 radiocarbon years ago." While this extended range for the AUP materials at Meadowcroft may be imprecise, the stratigraphic relationships at the site clearly indicate they pre-date the CPT period.

In addition to the lithic remains from lower Stratum IIa Adovasio et al. (*24*) also report the discovery of 14 "firepits," one "fire floor," two lithic/bone concentrations, and a single storage/refuse pit in the earliest deposits (Fig. S3). While hearths are often not considered "artifacts" they are themselves definitive evidence of an AUP occupation of the rockshelter.

Gault and Friedkin

The Gault and Debra L. Friedkin localities are open sites located along a small spring-fed stream on the ecotone between the central Texas hill country of the Edwards Plateau and the

Blackland Prairie on the adjacent coastal plains (Figs. S1 & S4) (*25, 28*). In addition to the water source, large quantities of high-quality Edwards chert attracted foragers to the site throughout the entire history of human occupation in the Americas. The overlapping residential base camps associated with these repeated visits are scattered along several kilometers of Buttermilk Creek below the headwater spring. Overall, the entire pre-contact sequence, from the AUP to Late Prehistoric periods, is present at closely-spaced localities along the creek margin. However, at any one locality the sequence may appear to be more episodic due to the shift in residential locations through time. For example, AUP occupations at the Friedkin locality (30), located only a few hundred meters downstream from Area 15 at the Gault site, date via OSL to between ~15,500-13,500 cal BP, and fill in a gap between the CPT and earlier AUP occupations evident at the Area 15 location.

Area 15 consisted of 6 x 8 m block excavation with the central 4 x 3 m block excavated to bedrock. Ten intact stratigraphic units underlying a disturbed mid-Archaic to Late Prehistoric midden were identified and matched to five AUP to mid-Archaic cultural horizons containing diagnostic artifacts (Fig. S5). More than 150,000 AUP artifacts, termed the "Gault Assemblage" by Williams et al. (*25, 26*), were recovered from the lowest two stratigraphic units (Units 1-2) above bedrock. These were separated from a similar number of CPT artifacts in unit 4 by a 10-15 cm thick zone of markedly reduced lithic debris in Unit 3.

Groundwater leaching of carbon throughout the site precluded ^{14}C dating, but the chronology is controlled by 21 single aliquot OSL age estimates from Area 15. OSL age estimates from CPT and younger cultural horizons match the known ages of cultural diagnostics in those horizons (*25*). Gault Assemblage materials are found within the lowest two stratigraphic layers deposited sometime between ~21,9700-~16,700 cal. The period of limited occupational intensity between the AUP and CPT occurred between ~16,700-~13,500 cal BP, with the following Clovis assemblage deposited sometime between ~13,500-~12,900 cal BP. Critics of the site (*116, 117*) have suggested that the stratigraphic integrity of the Debra L. Friedkin and Gault sites is questionable. This view is challenged by the stratigraphic sequence of projectile points in the deposits at the Gault site, as well as the presence of small notching flakes associated with Andice point production in the Middle Archaic that are not found in the lower units of the site (*25*). Research on the deposits from the Gault site are ongoing but early analysis indicates there is little to no movement of any materials larger than 1 cm.

Cooper's Ferry/Nipéhe

The Cooper's Ferry/Nipéhe site is located along the lower Salmon River in west-central Idaho at the confluence with the Rock Creek tributary canyon (Figs. S1 & S6-S8). The site was first test excavated in the 1960s by B. Robert Butler (*118*) and again in 1997 by Davis (*32-34*), with major work conducted within two excavation blocks between 2009 and 2018 (*31, 35, 36*). Geoarchaeological study of the site defined a stratified sequence of deposits, including a lower loess deposit (LU3/LUB3) that contains the AUP occupation. In addition to the recovery of lithic, bone, and shell artifacts, hearths and pit features were identified and excavated. Pit features originating wholly within LU3 and LUB3 or from its surface contained bifacially flaked stemmed projectile points. In some cases, these pits appear to represent point caches (*32-35*). Chronological controls for the age of the LU3/LUB3 depositions deposits are provided by 11 ^{14}C age estimates from Area A and seven from Area B. Dates on from a hearth within upper LU3 in Area A have a median average of ~14,600 cal BP, with the oldest dated samples from LU3 falling with a range of ~15,900-~14,200 cal BP. Bayesian modeling of the LU3 ^{14}C dates from Area A suggest human occupation of that part of Cooper's Ferry/Nipéhe began between ~16,560 and ~15,280 cal BP (*31*). Seven AMS dates on animal bone fragments from three pit features buried in Area B's LUB3 loess returned ages averaging ~15,785 cal BP (*36*). Lithic debitage and stemmed projectile points recovered *in situ* outside and stratigraphically below these Area B pit features indicates an older as yet undated human occupation. Critics of the proposed pre-Clovis occupation at the site have questioned its contextual integrity, arguing that vertical patterns in artifact quantities serve as a proxy for stratigraphic mixing (*117*). However, the claim that early cultural deposits at Cooper's Ferry/Nipéhe must be mixed is contradicted by multiple lines of geoarchaeological evidence, including intact pedogenic horizons, undisturbed cultural features (such as human-made pits and a hearth), and stratigraphically consistent radiocarbon ages on bone and charcoal that increase with depth. A more parsimonious explanation for the vertical distribution of cultural materials within the LU3/LUB3 deposits is that humans reoccupied the site frequently enough to match the pace of sediment accumulation.

Page-Ladson

The Page-Ladson site is contained in a sinkhole along the Aucilla River in the Florida panhandle (Fig. S1). Underwater excavations at the site from 1983-1997 and 2012-2014

identified a series of seven undisturbed stratigraphic units in the sinkhole (*37, 38*). AUP materials pre-dating and underlying artifacts related to the CPT era were recovered from units 3 and 4 in association with disarticulated mastodon bones. Cut marks and crushing suggests the mastodon was butchered. The earlier excavations produced seven ^{14}C dates associated with the earliest cultural deposits in Unit 3 ranging between ~14,600 and ~14,200 cal BP (*37*). Comprehensive dating of the depositional sequence associated with the later excavations produced 71 ^{14}C age estimates on wood preserved in the submerged deposits, with 24 of these coming from a stratigraphic column bracketing lithic materials and seven directly related to a biface and other flake tools (*37*). These seven have an average age of ~14,550 cal BP. A small collection of lithic remains and two possible bone tools appears to be associated with the mastodon butchering.

Schaefer and Hebior Mammoth

Schaefer and the nearby Hebior site are two mammoth (*Mammuthus primigenius*) butchering sites in southeastern Wisconsin located on the margin of what was the fluctuating Laurentide ice sheet at the time the bones were deposited (Fig. S1). Although the lithic assemblages at the two sites are limited, we include Schaefer here because the site is well dated, the lithics are in clear association with the mammoth bones, and the production and use of blade flakes at the site is consistent with one of the central features of the AUP lithic technology. The mammoths at both sites were found in topographic lows between north-south trending end moraines (*39*) where a series of melt-water lakes and ponds formed during the deglacial period (*40*). At the Schaefer site the bones were encased in stratified peats deposited under low-energy conditions and were lying on the surface of gray silty lacustrine clays. There was no evidence of bioturbation. The age of the mammoth is controlled by 14 ^{14}C dates directly on mammoth bone and another 16 on wood macrofossils recovered from the peat in and around the bones (*40*). The bone dates average about 14,750 cal BP with a similar average age for the supporting wood dates.

Taphonomic analysis of the mammoths suggests they were butchered (*41*). At Schaefer there are multiple cut and wedge marks on ten skeletal elements and the disarticulated bones were left in a pile, suggesting human intervention rather than trampling and/or carnivore disturbance. While there is some evidence of human or non-human trampling at the site, two

blade flakes were recovered from below the innominate, a position that cannot be explained by later deposition.

Paisley Caves

The Paisley Caves are a set of small closely-spaced caves overlooking Summer Lake in southeastern Oregon (Fig. S1). The Summer Lake basin is a sub-basin of the Pleistocene Lake Chewaucan system and was first exposed as the paleolake began to recede at the end of the LGM. When the caves were first occupied, they overlooked a broad, gently sloping, and probably grassy, plain leading down to the lake margin. The caves were first excavated in the late l930s when the remains of extinct Pleistocene megafauna were found on a living floor in Cave 3 in association with an assemblage of obsidian artifacts and debitage (*119, 120*). Doubts about the association remained until Dennis Jenkins and colleagues returned to the site and conducted additional excavations of the dry stratified deposits in Caves 2 and 5 beginning in 2002 and continuing for most of the next two decades (*44, 45, 121*). During that period, they obtained more than 400 ^{14}C age estimates from the caves, with more than 100 dating to earlier than ~12,800 cal BP. Many of these were run directly on human fecal remains found in Cave 5. The five earliest coprolites are dated to between ~12,500-~12,000 ^{14}C BP (~14,500-~14,000 cal BP) (*44*). That these early coprolites are indeed human has been confirmed through DNA analysis (*45*) and the presence of human fecal biomarkers (*122*).

In addition to the association with such Pleistocene megafauna as horse and camel, four obsidian stemmed point fragments, a biface, a flake tool, two hammerstones, and more than 250 pieces of debitage were recovered from the earliest levels in Caves 2 and 5 (levels LU1 and LU2). Jenkins et al. (*44*) provide the following bracketing ages for the four stemmed point fragments: 10,200±35 BP-10,855±30 BP (11,950-11,750 cal BP to 12,830-12,740 cal BP at 2σ), 10,855±30 BP-11,070±25 BP (12,830-12,740 cal BP to 13,090-12,920 cal BP at 2σ), 10,965±50 BP-12,140±70 BP (13,060-13,025 cal BP to 14,210-13,800 cal BP at 2σ), and 11,070±25 BP-11,340±50 BP (13,090-12,920 cal BP to 13,315-13,160 cal BP at 2σ). While all four could fall within the age range of the CPT (*3*), the older two may be as old, or older, than any directly dated Clovis diagnostics.

White Sands Footprints

A series of human footprints have been identified in a stratified set of shoreline muds adjacent Pleistocene Lake Otero in the Tularosa Basin of southcentral New Mexico (Fig. S1). These human footprints are associated with those of extinct megafauna such as ground sloths (*Folivora* sp.), mammoths (*Proboscidea* sp.), and camels and other bovids (*Cetartiodactyla* sp.) (*12*). The chronology of these footprints was initially controlled by eleven ^{14}C age estimates run on the fruits of *Ruppia cirrhosa* which were stratigraphically interleaved with the foot imprints, and which produced age estimates of ~23,000 to 21,000 cal BP (*12*). However, *Ruppia* sp. is an aquatic plant which draws much of the carbon it employs in its growth from dissolved inorganic carbonates in the water. As a result, in can, and usually does, produce ^{14}C age estimates which are older than their true ages (e.g., *13*). In the Tularosa Basin modern *Ruppia cirrhosa* fruits have been dated to as old as about 7350 cal BP (~7275–7425 cal BP, 2σ range), suggesting the footprints may actually date to after the Last Glacial Maximum (*14*).
This criticism was addressed by obtaining additional ^{14}C age estimates on conifer pollen extracted from the sediments enclosing the footprints and Optically Stimulated Luminescence age estimates on sand from the same units (*10*). These produced age estimates which are broadly correlative with the *Ruppia* dates, supporting the initial chronological assessments. Yet, even the reliability of those estimates has been questioned (e.g., *14*), and the debate continues (e.g., *11, 15*). Regardless of the final outcome of this debate two important points are critical: 1) There has been no question that the footprints were made by a group of humans ranging in age from toddlers to adults, and that these human footprints were associated with the footprints of extinct Pleistocene megafauna; and 2) Even if the footprints do prove to be as much as ~3000 to ~7000 years younger than first estimated, and the jury is still out on that question, they would still date to somewhere in the range of ~16,000 to ~20,000 cal BP, or as old or older than the other sites we discuss here.

Provenance

This article is a review and does not present unpublished data, nor does it involve the collection, analysis, or curation of original fossils, artifacts, ecofacts, or human remains. All specimens and datasets discussed herein have been previously reported in the published literature or are currently in press. Information regarding the original circumstances of collection, including where, when, and by whom materials were collected, is available in the cited primary

sources. Likewise, authentication and dating of the materials were carried out by the original researchers and are described in those same works. Researchers seeking to examine these materials should consult the cited publications and contact the original authors or curating institutions to request access or additional information.

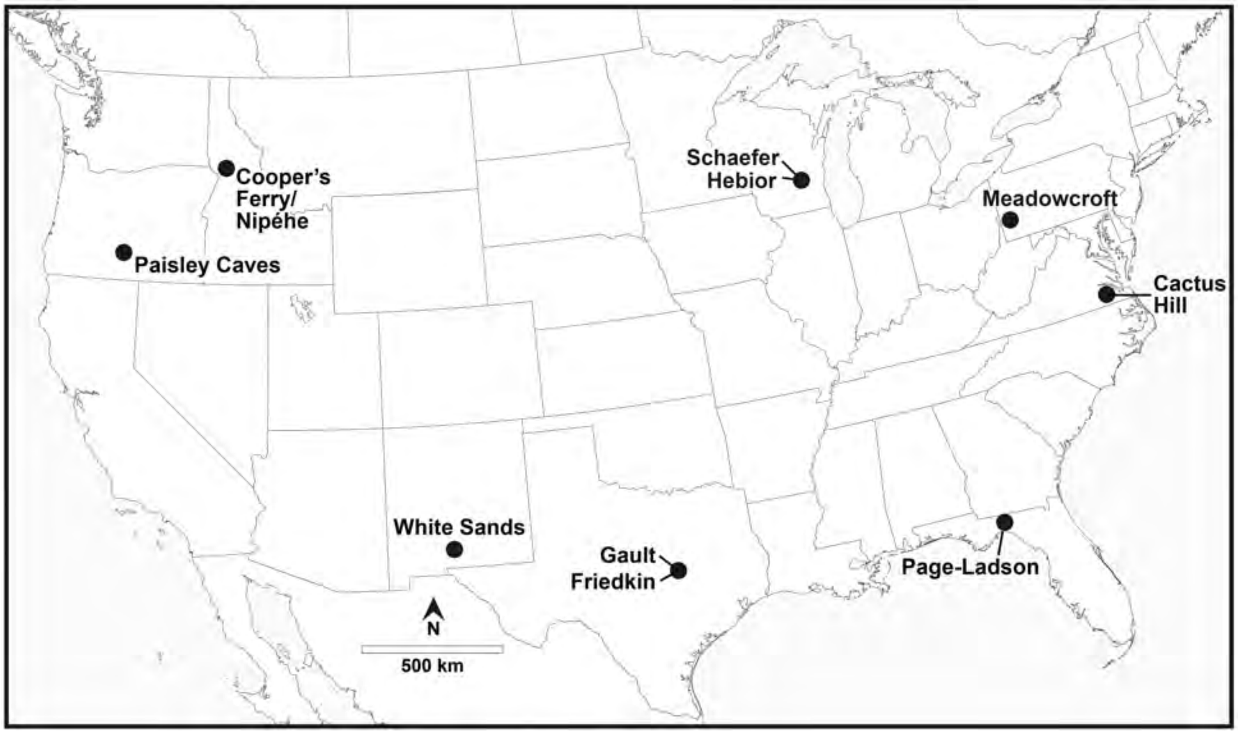

Fig. S1 – Site Locations. Location of major North American Upper Paleolithic sites discussed in the text.

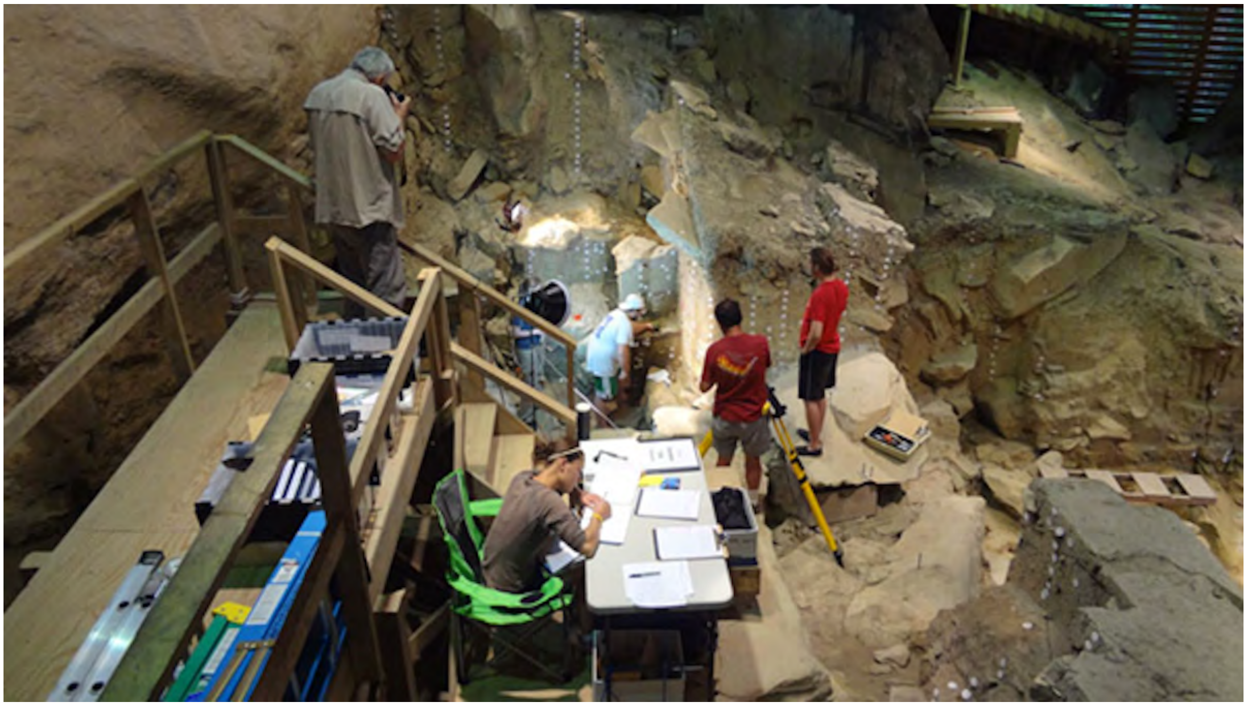

Fig. S2 – Meadocroft Rockshelter Interior. Meadowcroft Rockshelter, western Pennsylvania, during excavation. Photo courtesy of James M. Adovasio.

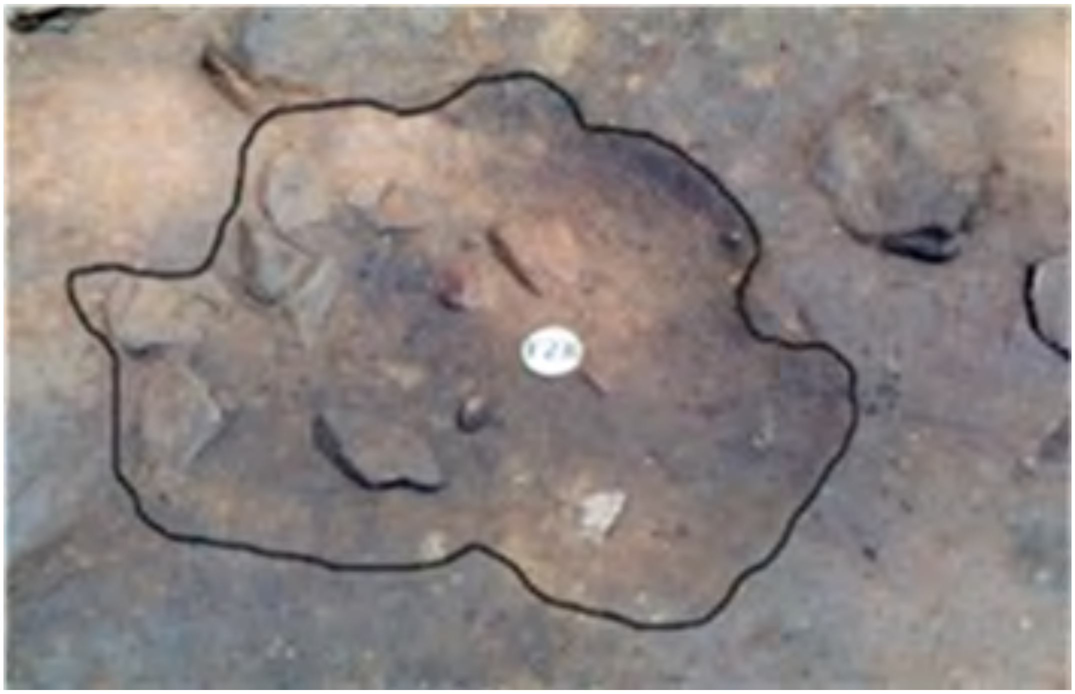

Fig. S3 -Hearth Feature at Meadowcroft Rockshelter. View of Hearth F276 on a living surface in lower Stratum IIa at Meadowcroft Rockshelter radiocarbon dated to 13,270±340 ^{14}C BP, or ~16,000 cal BP (17,025-14,970 cal BP at 2σ). Photo courtesy of James M. Adovasio.

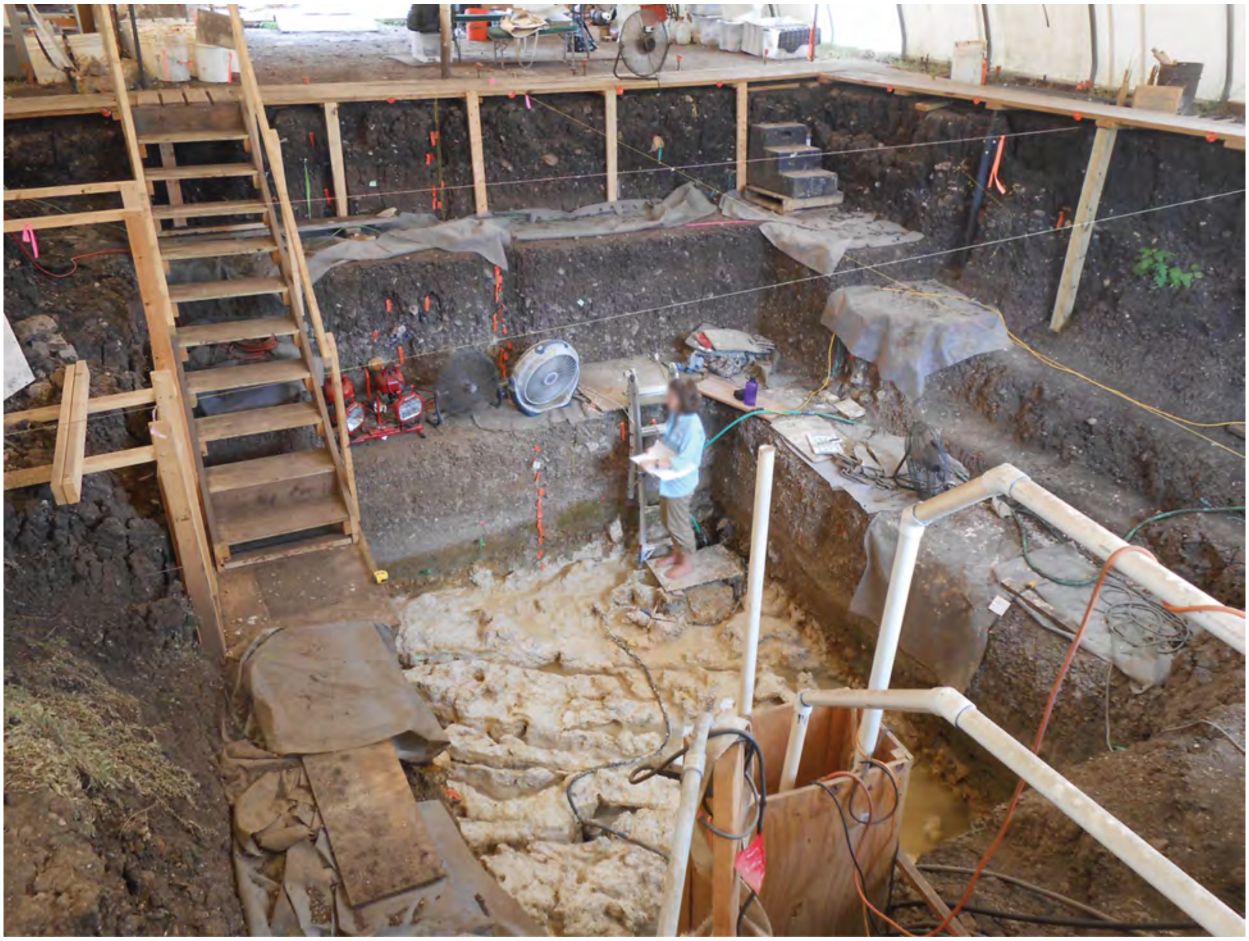

Fig. S4 - Area 15 at the Gault Site, Central Texas. Area 15 showing excavations taken to bedrock. Photo courtesy of Thomas Williams.

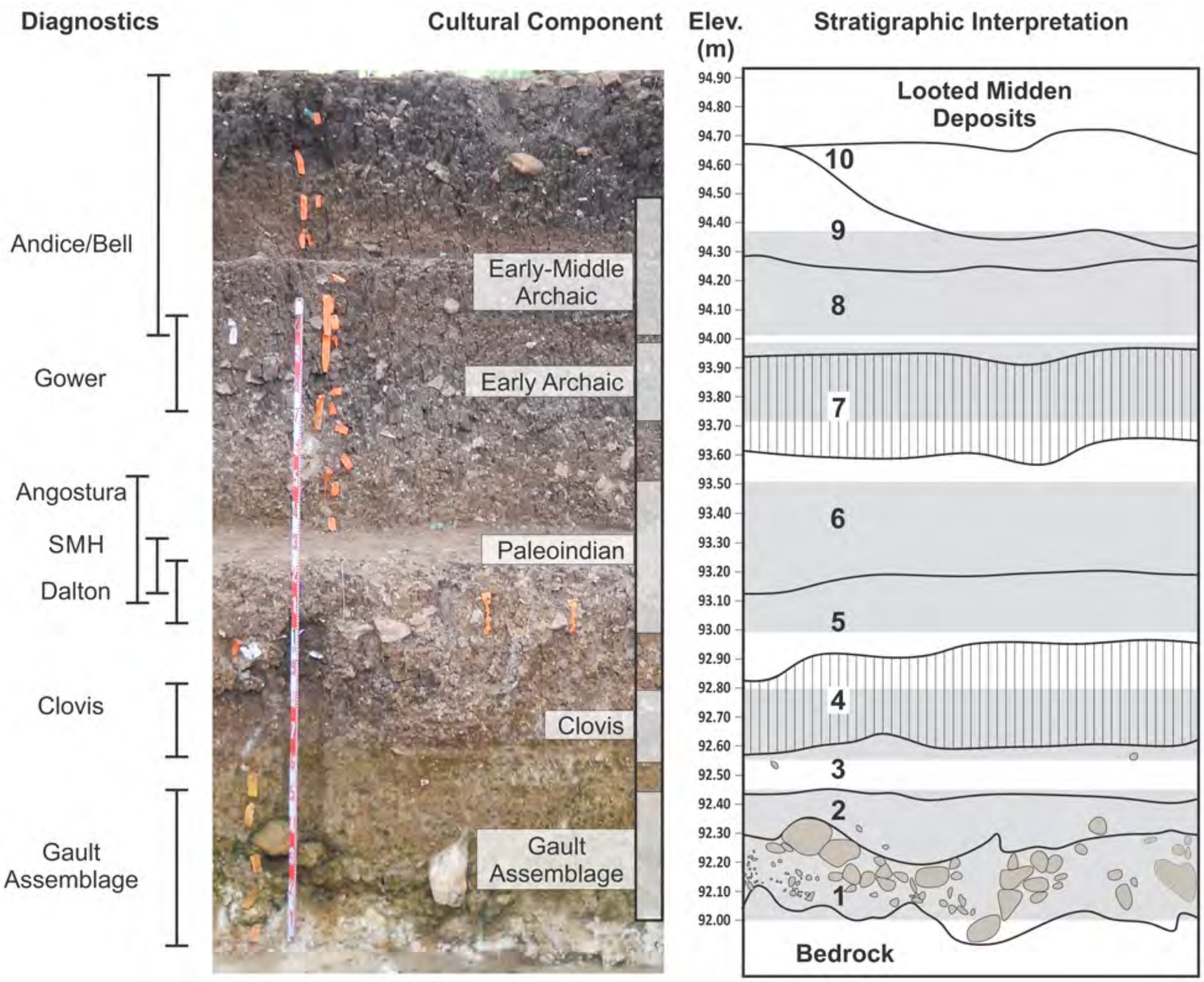

Fig. S5 – Stratigraphic Sequence at the Gault Site. Stratigraphic sequence in the Area 15 excavation block at the Gault site showing the relationship between the Gault Assemblage deposits and overlying diagnostic cultural materials (SMH = St. Mary's Hall). Reproduced from Williams, T. J. et al. (25), Science Advances, https://doi.org/10.1126/sciadv.aar5954 (2018), AAAS. RightsLink License No. 609052136540.

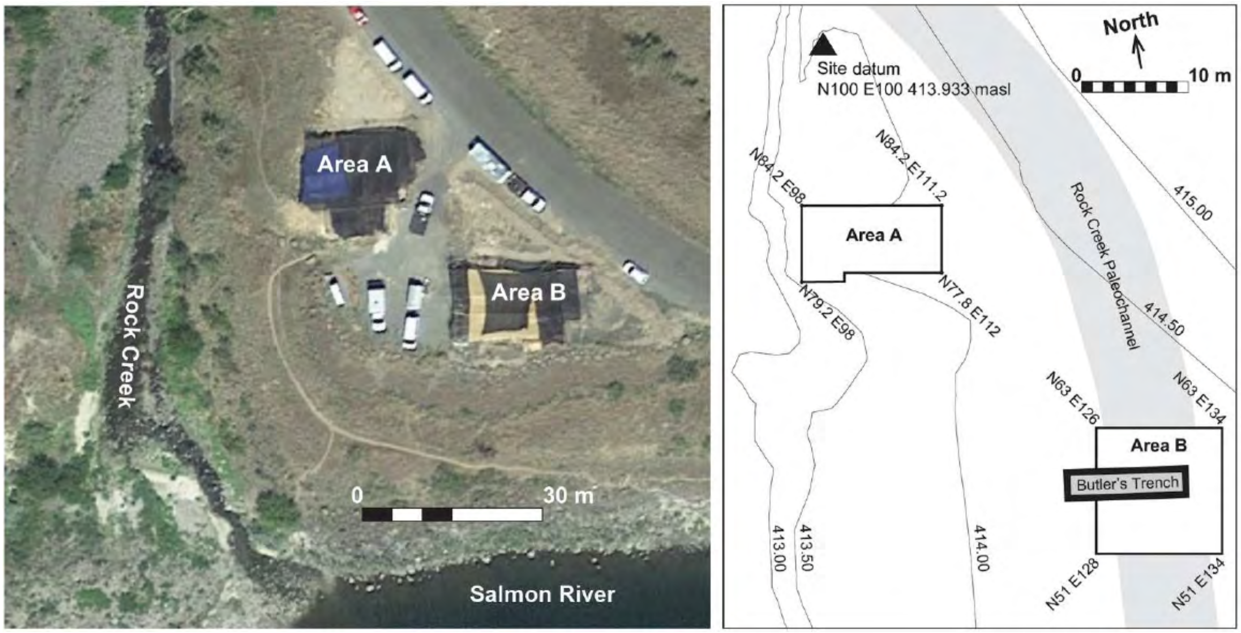

Fig. S6 – Aerial View of the Cooper's Ferry/Nipéhe Site. Aerial view of the Cooper's Ferry/Nipéhe site the locations of excavation areas A and B at the confluence of Rock Creek and the Salmon River in western Idaho. Modified from Davis, L.G. et al. (36), Science Advances, https://doi.org/10.1126/sciadv.abq1130 (2022), AAAS. Published under the Creative Commons Attribution License (CC BY 4.0).

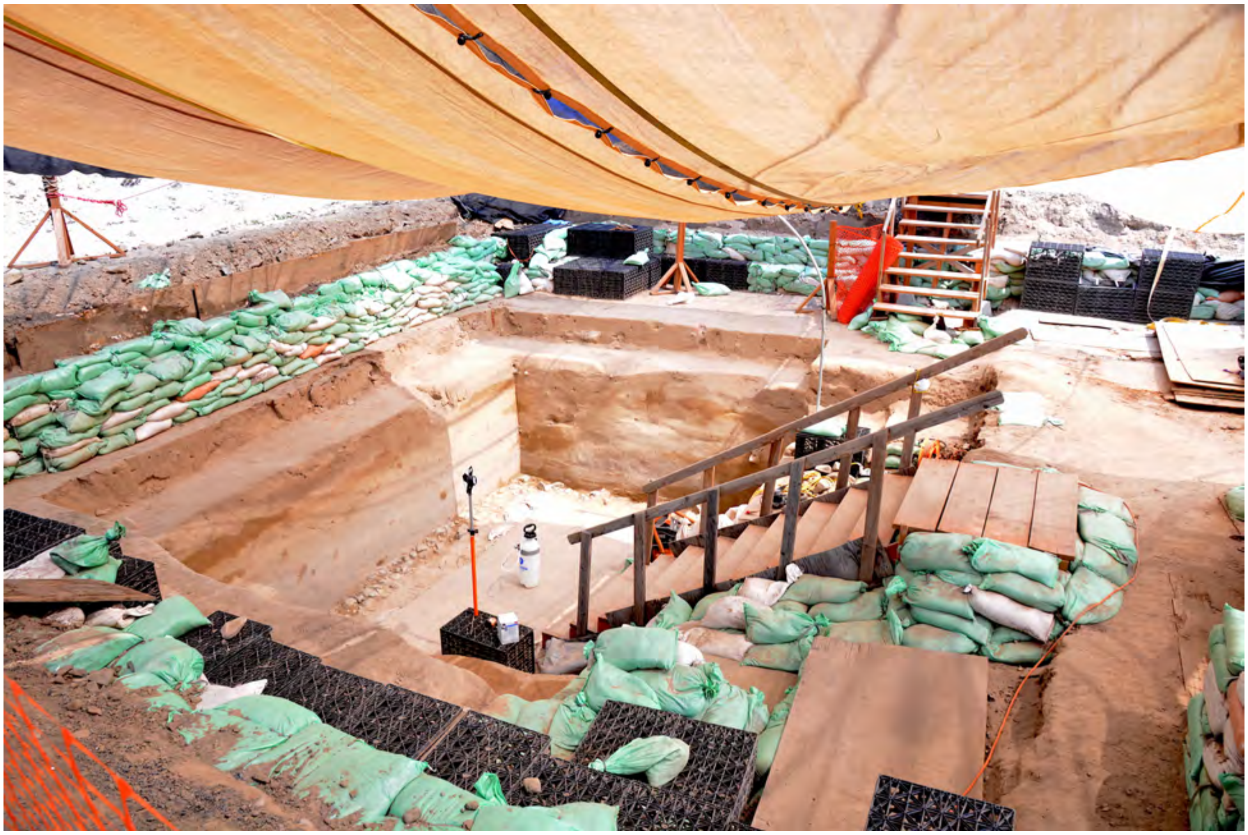

Fig. S7 - Overview of Area B at the Cooper’s Ferry/Nipéhe Site. Photo showing excavations down to basal gravel deposits. Image courtesy of Loren Davis.

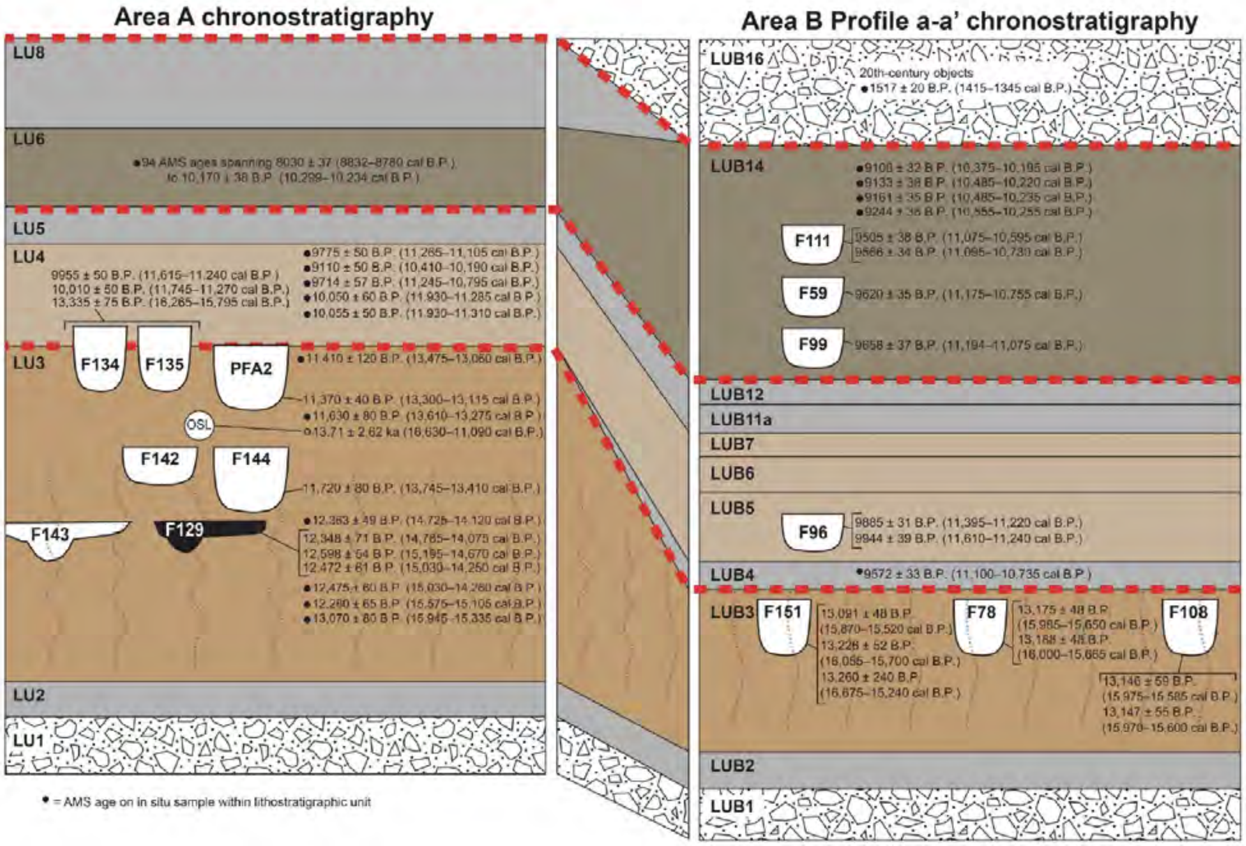

Fig. S8 - Chronostratigraphic Model of the Cooper’s Ferry/Nipéhe Site. Model correlating Area A and Area B deposits and cultural features. Red dashed lines mark unconformities at eroded paleosurfaces. The vertical scale of each composite profile is ~3.0 m. Modified from Davis, L. G. et al. (36), Science Advances, https://doi.org/10.1126/sciadv.abq1130 (2022), AAAS. Published under the Creative Commons Attribution License (CC BY 4.0).

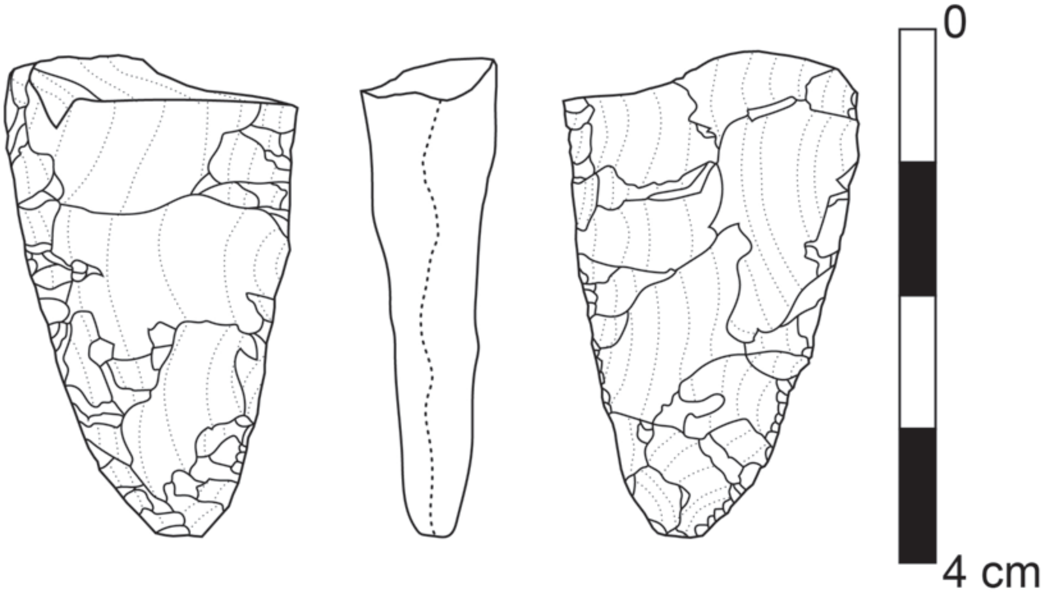

Fig. S9 - Illustration of a Stemmed Point Base from Paisley Caves. Stemmed point base found *in situ* at Paisley Caves, Cave 5, bracketed between ^{14}C ages of 11,070±25 BP-11,340±50 BP (13,090-12,920 cal BP to 13,315-13,160 cal BP at 2σ). Modified from Jenkins, D. L. et al. (44), Science, https://doi.org/10.1126/science.1218443 (2012), AAAS. RightsLink License No. 6093280864935.

REFERENCES AND NOTES

1. M. B. Collins, D. J. Stanford, D. L. Lowery, B. A. Bradley, "North America before Clovis: Variance in temporal/spatial cultural patterns, 27,000–13,000 cal yr BP," in *Paleoamerican Odyssey*, K. E. Graf, C. V. Ketron, M. R. Waters, Eds. (Center for the Study of the First Americans, Texas A&M Univ., 2013), pp. 521–539.

2. B. Buchanan, J. D. Kilby, J. M. LaBelle, T. A. Surovell, J. Holland-Lulewicz, M. J. Hamilton, Bayesian modeling of the Clovis and Folsom radiocarbon records indicates a 200-year multigenerational transition. *Am. Antiq.* **87**, 567–580 (2022).

3. R. L. Rosencrance, K. N. McDonough, G. M. Smith, C. S. Jazwa, D. G. Duke, D. L. Jenkins, J. Clements, D. O. Stueber, L. S. Henrikson, Bayesian analysis of Haskett projectile point radiocarbon dates in the Intermountain West demonstrates contemporaneity with Clovis and Folsom points. *PaleoAmerica* **10**, 132–155 (2024).

4. T. J. Williams, D. B. Madsen, The New World Upper Paleolithic. *PaleoAmerica* **6**, 4–22 (2020).

5. L. G. Davis, D. B. Madsen, The coastal migration theory: Formulation and testable hypotheses. *Quat. Sci. Rev.* **249**, 106605 (2020).

6. D. J. Meltzer, *The Great Paleolithic War: How Science Forged an Understanding of America's Ice Age Past* (University of Chicago Press, 2019).

7. J. F. Hoffecker, S. A. Elias, D. H. O'Rourke, G. R. Scott, N. H. Bigelow, Beringia and the global dispersal of modern humans. *Evol. Anthropol.* **25**, 64–78 (2016).

8. B. A. Potter, J. F. Baichtal, A. B. Beaudoin, L. Fehren-Schmitz, C. V. Haynes, V. T. Holliday, C. E. Holmes, J. I. Ives, R. L. Kelly, B. Llamas, R. S. Malhi, D. S. Miller, D. Reich, J. D. Reuther, S. Schiffels, T. A. Surovell, Current evidence allows multiple models for the peopling of the Americas. *Sci. Adv.* **4**, eaat5473 (2018).

9. M. R. Waters, Late Pleistocene exploration and settlement of the Americas by modern humans. *Science* **365**, eaat5447 (2019).

10. J. S. Pigati, K. B. Springer, J. S. Honke, D. Wahl, M. R. Champagne, S. R. H. Zimmerman, H. J. Gray, V. L. Santucci, D. Odess, D. Bustos, M. R. Bennett, Independent age estimates resolve the controversy of ancient human footprints at White Sands. *Science* **382**, 73–75 (2023).

11. V. T. Holliday, J. D. Windingstad, J. Bright, B. G. Phillips, J. B. Butler, R. Breslawski, J. E. Bowman, Paleolake geochronology supports Last Glacial Maximum (LGM) age for human tracks at White Sands, New Mexico. *Sci. Adv.* **11**, eadv4951 (2025).

12. M. R. Bennett, D. Bustos, J. S. Pigati, K. B. Springer, T. M. Urban, V. T. Holliday, S. C. Reynolds, M. Budka, J. S. Honke, A. M. Hudson, B. Fenerty, C. Connelly, P. J. Martinez, V. L. Santucci, D. Odess, Evidence of humans in North America during the last glacial maximum. *Science* **373**, 1528–1531 (2021).

13. G. Oviatt, D. B. Madsen, D. Rhode, L. G. Davis, A critical assessment of claims that human footprints in the Lake Otero basin, New Mexico date to the Last Glacial Maximum. *Quatern. Res.* **111**, 138–147 (2023).

14. D. Rhode, C. M. Neudorf, D. Rachal, L. G. Davis, D. B. Madsen, R. Dello-Russo, Unresolved: Persistent problems with the White Sands locality 2 geochronology. *PaleoAmerica* **10**, 10–27 (2024).

15. D. M. Rachal, R. Dello-Russo, M. Cuba, The Pleistocene footprints are younger than we thought: Correcting the radiocarbon dates of *Ruppia* seeds, Tularosa Basin, New Mexico. *Quat. Res.* **117**, 67–78 (2024).

16. D. B. Madsen, A framework for the initial occupation of the Americas. *PaleoAmerica* **1**, 217–250 (2015).

17. K. E. Graf, I. Buvit, Human dispersal from Siberia to Beringia: Assessing a Beringian standstill in light of the archaeological evidence. *Curr. Anthro.* **58**, S583–S603 (2017).

18. A. M. Younie, T. Goebel, E. Combs, Chindadn bifaces and the archaeology of Terminal-Pleistocene Alaska. *PaleoAmerica* **9**, 364–383 (2023).

19. J. M. McAvoy, L. D. McAvoy, *Cactus Hill and Other Excavated Sites*, Nottoway River Survey Research Report No. 5, (Sandston, Virginia, 2015).

20. R. I. Macphail, J. M. McAvoy, A micromorphological analysis of stratigraphic integrity and site formation at Cactus Hill, an early Paleoindian and hypothesized pre-Clovis occupation in south-central Virginia USA. *Geoarch. Int. J.* **23**, 675–694 (2008).

21. J. M. Adovasio, J. Donahue, R. Stuckenrath, The Meadowcroft Rockshelter radiocarbon chronology 1975–1990. *Am. Antiq.* **55**, 348–354 (1990).

22. J. M. Adovasio, R. C. Carlisle, The Meadowcroft Rockshelter. *Science* **239**, 713–714 (1988).

23. J. M. Adovasio, D. R. Pedler, J. Donahue, R. Stuckenrath, Two decades of debate on Meadowcroft Rockshelter. *N. Am. Archaeol.* **19**, 317–341 (1998).

24. J. M. Adovasio, J. D. Gunn, J. Donahue, R. Stuckenrath, Meadowcroft Rockshelter, 1977: An overview. *Am. Antiq.* **43**, 632–651 (1978).

25. T. J. Williams, M. B. Collins, K. Rodrigues, W. J. Rink, N. Velchoff, A. Keen-Zebert, A. Gilmer, C. D. Frederick, S. J. Ayala, E. R. Prewitt, Evidence of an early projectile point technology in North America at the Gault Site, Texas, USA. *Sci. Adv.* **4**, eaar5954 (2018).

26. T. J. Williams, N. Velchoff, M. B. Collins, B. A. Bradley, "Stone tool technology at the Gault Site," in *People and Culture in Ice Age Americas: New Dimensions in PaleoAmerican* Archaeology, R. Suarez, C. F. Ardelean, Eds. (University of Utah Press, 2019), pp. 134–155.

27. M. B. Collins, *Clovis Blade Technology* Texas Archaeology and Ethnohistory Series (University of Texas Press, Austin, 1999).

28. M. R. Waters, S. L. Forman, T. A. Jennings, L. C. Nordt, S. G. Driese, J. M. Feinberg, J. L. Keene, J. Halligan, A. Lindquist, J. Pierson, C. T. Hallmark, M. B. Collins, J. E. Wiederhold, The Buttermilk Creek complex and the origins of clovis at the Debra L. Friedkin site, Texas. *Science* **331**, 1599–1603 (2011).

29. T. Jennings, M. R. Waters, Pre-Clovis lithic technology at the Debra L. Friedkin site, Texas: Comparisons to Clovis through site-level behavior, technological trait-list, and cladistic analyses. *Am. Ant.* **79**, 25–44 (2014).

30. M. R. Waters, J. L. Keene, S. L. Forman, E. R. Prewitt, D. L. Carlson, J. E. Wiederhold, Pre-Clovis projectile points at the Debra L. Friedkin site, Texas—Implications for the Late Pleistocene peopling of the Americas. *Sci. Adv.* **4**, eaat4505 (2018).

31. L. G. Davis, D. B. Madsen, L. Becerra-Valdivia, T. Higham, D. A. Sisson, S. M. Skinner, D. Stueber, A. J. Nyers, A. Keen-Zebert, C. Neudorf, M. Cheyney, M. Izuho, F. Iizuka, S. R. Burns, C. W. Epps, S. C. Willis, I. Buvit, Late Upper Paleolithic occupation at Cooper's Ferry, Idaho, USA, ~16,000 years ago. *Science* **365**, 891–897 (2019).

32. L. G. Davis, D. A. Sisson, An early stemmed point cache from the lower Salmon River canyon of West-Central Idaho. *Curr. Res. Pleistocene* **15**, 12–14 (1998).

33. L. G. Davis, C. E. Schweger, Geoarchaeological context of Late Pleistocene and Early Holocene occupation at the Cooper's Ferry site, Western Idaho, USA. *Geoarch. Int. J.* **19**, 685–704 (2004).

34. L. G. Davis, A. J. Nyers, S. C. Willis, Context, provenance and technology of a Western Stemmed Tradition artifact cache from the Cooper's Ferry site, Idaho. *Am. Antiq.* **79**, 596–615 (2014).

35. L. G. Davis, D. W. Bean, A. J. Nyers, Morphometric and technological attributes of Western Stemmed Tradition projectile points revealed in a second artifact cache from the Cooper's Ferry site, Idaho. *Am. Antiq.* **82**, 536–557 (2017).

36. L. G. Davis, D. B. Madsen, D. A. Sisson, L. Becerra-Valdivia, T. Higham, D. Stueber, D. W. Bean, A. J. Nyers, A. Carroll, C. Ryder, M. Sponheimer, Dating of a large tool assemblage at the Cooper's Ferry site (Idaho, USA) to ~15,785 cal yr BP extends the age of stemmed points in the Americas. *Sci. Adv.* **8**, eade1248 (2022).

37. J. J. Halligan, M. R. Waters, A. Perrotti, I. J. Owens, J. M. Feinberg, M. D. Boume, B. Fenerty, B. Winsborough, D. Carlson, D. C. Fisher, T. W. Stafford Jr., J. S. Dunbar, Pre-Clovis occupation 14,550 years ago at the Page-Ladson site, Florida, and the peopling of the Americas. *Sci. Adv.* **2**, e1600375 (2016).

38. S. D. Webb, *First Floridians and Last Mastodons: The Page-Ladson Site in the Aucilla River*, Topics in Geobiology (Springer, 2006).

39. D. F. Overstreet, M. F. Kolb, Geoarchaeological contexts for Late Pleistocene archaeological sites with human-modified woolly mammoth remains in southeastern Wisconsin, U.S.A. *Geoarch. Int. J.* **18**, 91–114 (2003).

40. D. J. Joyce, Chronology and new research on the Schaefer mammoth (?*Mammuthus primigenius*) site, Kenosha County, Wisconsin, USA. *Quat. Int.* **142**, 44–57 (2006).

41. E. Johnson, Along the ice margin—The cultural taphonomy of Late Pleistocene mammoth in southeastern Wisconsin (USA). *Quat. Int.* **169-170**, 64–83 (2007).

42. D. F. Overstreet, Still more on cultural contexts of mammoth and mastodont in the southwestern Lake Michigan Basin. *Curr. Res. in the Pleist.* **13**, 36–38 (1996).

43. D. F. Overstreet, Late Pleistocene geochronology and the Paleo-Indian penetration of the Southwestern Lake Michigan Basin. *Wisconsin Archeol.* **79**, 28–52 (1998).

44. D. L. Jenkins, L. G. Davis, T. W. Stafford Jr., P. F. Campos, B. Hockett, G. T. Jones, L. S. Cummings, C. Yost, T. J. Connolly, R. M. Yohe, S. C. Gibbons, Clovis age Western Stemmed projectile points and human coprolites at the Paisley Caves. *Science* **337**, 223–228 (2012).

45. D. L. Jenkins, L. G. Davis, T. W. Stafford, P. F. Campos, T. L. Connolly, L. S. Cummings, M. Hofreiter, B. Hockett, K. McDonough, I. Luthe, P. W. O'Grady, "Geochronology, archaeological context, and DNA at the Paisley Caves," in *Paleoamerican Odyssey.* K. E. Graf, C. V. Ketron, M. R. Waters Eds., Center for the Study of the First Americans (Texas A&M University, 2013), pp. 521–539.

46. A. L. Bryan, "The stemmed point tradition: An early technological tradition in western North America," in *Anthropological Papers in Memory of Earl H. Swanson, Jr.*, L. Harten, C. Warren, D. Tuohy, Eds. (Spec. Publ. Idaho State University Mus. Nat. Hist., Pocatello, 1980), pp. 77–107.

47. A. L. Bryan, "The relationship of the stemmed point and fluted point traditions in the Great Basin," in *Early Human Occupation in Far Western North America: The Clovis–Archaic Interface*, J. A. Willig, C. M. Aikens, J. L. Fagan, Eds., Nevada State Museum Anthropological Papers No. 21 (Nevada State Museum, 1988), pp. 53–74.

48. C. Beck, G. T. Jones, Clovis and Western Stemmed: Population migration and the meeting of two technologies in the Intermountain West. *Am. Antiq.* **75**, 81–116 (2010).

49. D. B. Madsen, D. N. Schmitt, D. Page, *The Paleoarchaic Occupation of the Old River Bed Delta*, University of Utah Anthropological Papers 128 (University of Utah Press, 2015).

50. D. J. Stanford, B. Bradley, *Across Atlantic Ice: The Origin of America's Clovis Culture* (University of California Press, 2012).

51. E. Willerslev, D. J. Meltzer, Peopling of the Americas as inferred from ancient genomics. *Nature* **594**, 356–364 (2021).

52. J. de Acosta, *The Natural and Moral History of the Indies*, reprinted from the English Translated Edition of Edward Grimston, C. R. Markham, Ed. (Hakluyt Society, 1604).

53. J. F. Hoffecker, S. A. Elias, O. Potapova, Arctic Beringia and native American origins. *PaleoAmerica* **6**, 158–168 (2020).

54. E. Tamm, T. Kivisild, M. Reidla, M. Metspalu, D. G. Smith, C. J. Mulligan, C. M. Bravi, O. Rickards, C. Martinez-Labarga, E. K. Khusnutdinova, S. A. Fedorova, M. V. Golubenko, V. A. Stepanov, M. A. Gubina, S. I. Zhadanov, L. P. Ossipova, L. Damba, M. I. Voevoda, J. E. Dipierri, R. Villems, R. S. Malhi, Beringian standstill and spread of Native American founders. *PLOS ONE* **2**, e829 (2007).

55. M. K. Faught, Where was the PaleoAmerind standstill? *Quat. Int.* **444**, 10–18 (2017).

56. T. Goebel, H. L. Smith, L. DiPietro, M. R. Waters, B. Hockett, K. E. Graf, R. Gal, S. B. Slobodin, R. J. Speakman, S. G. Driese, D. Rhode, Serpentine hot springs, Alaska: Results of excavations and implications for the age and significance of northern fluted points. *J. Arch. Sci.* **40**, 4222–4233 (2013).

57. J. V. Moreno-Mayar, L. Vinner, P. de Barros Damgaard, C. de la Fuente, J. Chan, J. P. Spence, M. E. Allentoft, T. Vimala, F. Racimo, T. Pinotti, S. Rasmussen, A. Margaryan, M. I. Orbegozo, D. Mylopotamitaki, M. Wooller, C. Bataille, L. Becerra-Valdivia, D. Chivall, D. Comeskey, T. Devièse, D. K. Grayson, L. George, H. Harry, V. Alexandersen, C. Primeau, J. Erlandson, C. Rodrigues-Carvalho, S. Reis, M. Q. R. Bastos, J. Cybulski, C. Vullo, F. Morello, M. Vilar, S. Wells, K. Gregersen, K. L. Hansen, N. Lynnerup, M. M. Lahr, K. Kjær, A. Strauss, M. Alfonso-Durruty, A. Salas, H. Schroeder, T. Higham, R. S. Malhi, J. T. Rasic, L. Souza, F. R. Santos, A.-S. Malaspinas, M. Sikora, R. Nielsen, Y. S. Song, D. J. Meltzer, E. Willerslev, Early human dispersals within the Americas. *Science* **362**, eaav2621 (2018).

58. M. Sikora, V. V. Pitulko, V. C. Sousa, M. E. Allentoft, L. Vinner, S. Rasmussen, A. Margaryan, P. de Barros Damgaard, C. de la Fuente, G. Renaud, M. A. Yang, Q. Fu, I. Dupanloup, K. Giampoudakis, D. Nogués-Bravo, C. Rahbek, G. Kroonen, M. Peyrot, H. McColl, S. V. Vasilyev, E. Veselovskaya, M. Gerasimova, E. Y. Pavlova, V. G. Chasnyk, P. A. Nikolskiy, P. S. Grebenyuk, A. Y. Fedorchenko, A. I. Lebedintsev, S. B. Slobodin, B. A. Malyarchuk, R. Martiniano, M. Meldgaard, L. Arppe, J. U. Palo, T. Sundell, K. Mannermaa, M. Putkonen, V. Alexandersen, C. Primeau, N. Baimukhanov, R. S. Malhi, K.-G. Sjögren, K. Kristiansen, A. Wessman, A. Sajantila, M. M. Lahr, R. Durbin, R. Nielsen, D. J. Meltzer, L. Excoffier, E. Willerslev, The population history of northeastern Siberia since the Pleistocene. *Nature* **570**, 182–188 (2019).

59. I. Buvit, K. Terry, Outside Beringia: Why the Northeast Asian upper paleolithic record does not support a long standstill model. *PaleoAmerica* **2**, 281–285 (2016).

60. I. Buvit, K. Terry, M. Izuho, "Pathways along the Pacific: Using early stone tools to reconstruct coastal migration between Japan and the Americas," in *Paleolandscapes and Archaeology:Lessons for the past and Future*, M. T. Carson, Ed. (Routledge, 2022), pp. 39–81.

61. M. Raghavan, M. Steinrücken, K. Harris, S. Schiffels, S. Rasmussen, M. De Giorgio, A. Albrechtsen, C. Valdiosera, M. C. Ávila-Arcos, A.-S. Malaspinas, A. Eriksson, I. Moltke, M. Metspalu, J. R. Homburger, J. Wall, O. E. Cornejo, J. V. Moreno-Mayar, T. S. Korneliussen, T. Pierre, M. Rasmussen, P. F. Campos, P. de Barros Damgaard, M. E. Allentoft, J. Lindo, E. Metspalu, R. Rodríguez-Varela, J. Mansilla, C. Henrickson, A. Seguin-Orlando, H. Malmström, T. Stafford Jr., S. S. Shringarpure, A. Moreno-Estrada, M. Karmin, K. Tambets, A. Bergström, Y. Xue, V. Warmuth, A. D. Friend, J. Singarayer, P. Valdes, F. Balloux, I. Leboreiro, J. L. Vera, H. Rangel-Villalobos, D. Pettener, D. Luiselli, L. G. Davis, E. Heyer, C. P. E. Zollikofer, M. S. Ponce de León, C. I. Smith, V. Grimes, K.-A. Pike, M. Deal, B. T. Fuller, B. Arriaza, V. Standen, M. F. Luz, F. Ricaut, N. Guidon, L. Osipova, M. I. Voevoda, O. L. Posukh, O. Balanovsky, M. Lavryashina, Y. Bogunov, E. Khusnutdinova, M. Gubina, E. Balanovska, S. Fedorova, S. Litvinov, B. Malyarchuk, M. Derenko, M. J. Mosher, D. Archer, J. Cybulski, B. Petzelt, J. Mitchell, R. Worl, P. J. Norman, P. J. Parham, B. M. Kemp, T. Kivisild, C. Tyler-Smith, M. S. Sandhu, M. Crawford, R. Villems, D. G. Smith, M. R. Waters, T. Goebel, J. R. Johnson, R. S. Malhi, M. Jakobsson, D. J. Meltzer, A. Manica, R. Durbin, C. D. Bustamante, Y. S. Song, R. Nielsen, E. Willerslev, Genomic evidence for the Pleistocene and recent population history of Native Americans. *Science* **349**, aab3884 (2015).

62. B. Llamas, L. Fehren-Schmitz, G. Valverde, J. Soubrier, S. Mallick, N. Rohland, S. Nordenfelt, C. Valdiosera, S. M. Richards, A. Rohrlach, M. I. B. Romero, I. F. Espinoza, E. T. Cagigao, L. W. Jiménez, K. Makowski, I. S. L. Reyna, J. M. Lory, J. A. B. Torrez, M. A. Rivera, R. L. Burger, M. C. Ceruti, J. Reinhard, R. S. Wells, G. Politis, C. M. Santoro, V. G. Standen, C. Smith, D. Reich, S. Y. W. Ho, A. Cooper, W. Haak, Ancient mitochondrial DNA provides high-resolution time scale of the peopling of the Americas. *Sci. Adv.* **2**, e1501385 (2016).

63. T. Pinotti, A. Bergström, M. Geppert, M. Bawn, D. Ohasi, W. Shi, D. R. Lacerda, A. Solli, J. Norstedt, K. Reed, K. Dawtry, F. González-Andrade, C. Paz-Y-Miño, S. Revollo, C. Cuellar, M. S. Jota, J. E. Santos Jr., Q. Ayub, T. Kivisild, J. R. Sandoval, R. Fujita, Y. Xue, L. Roewer, F. R. Santos, C. Tyler-Smith, Y chromosome sequences reveal a short Beringian standstill, rapid expansion, and early population structure of Native American founders. *Curr. Biol.* **29**, 149–157.e3 (2019).

64. Hokkaido Archaeological Operation Center, *Shirataki Isekigun VII: The Shirataki Group of Sites VII* (in Japanese) (Hokkaido Archaeological Operation Center, 2006).

65. M. Izuho, F. Akai, Y. Nakazawa, A. Iwase, "The upper paleolithic of Hokkaido: Current evidence and its geoarchaeological framework," in *Environmental Changes and Human Occupation in East Asia during OIS 3 and OIS 2*, A. Ono, M. Izuho Eds., BAR International Series 2352 (Archaeopress, 2012), pp. 109–128.

66. M. Izuho, D. Kunikita, Y. Nakazawa, N. Oda, K. Hiromatsu, O. Takahashi, New AMS dates from the Shukubai-Kaso site (loc. Sankakuyama), Hokkaido (Japan): Refining the chronology of small flake-based assemblages during the Early Upper Paleolithic in the Paleo-Sakhalin-Hokkaido-Kuril Peninsula. *PaleoAmerica* **4**, 134–150 (2018).

67. Makubetsu Town Board of Education (MTBE), *Satsunai N Site* (in Japanese) (Makubetsu Town Board of Education, 2000).

68. Obihiro City Board of Education (OCBE), *Obihiro Taisho Sites 2* (in Japanese) (Obihiro City Board of Education, 2006).

69. I. Buvit, M. Izuho, K. Terry, Radiocarbon dates, microblades and Late Pleistocene human migrations in the Transbaikal, Russia and the Paleo-Sakhalin-Hokkaido-Kuril Peninsula. *Quat. Int.* **100**, 100–119 (2016).

70. R. G. Elston, P. J. Brantingham, Microlithic technology in Northern Asia: A risk-minimizing strategy of the late paleolithic and early holocene. *Archaeol. Pap. Am. Anthropol. Assoc.* **12**, 103–116 (2002).

71. K. Morisaki, F. Iizuka, M. Izuho, M. Aldenderfer, More on mobility and sedentism: Changes in adaptation from Upper Paleolithic to Incipient Jomon, Tanegashima Island, southern Japan. *PLOS ONE* **20**, e0314311 (2025).

72. F. Iizuka, The timing and behavioral context of the Late-Pleistocene adoption of ceramics in greater East and Northeast Asia and the First People (without pottery) in the Americas. *PaleoAmerica* **4**, 267–324 (2018).

73. H. Dong, Z. Liu, H. Wu, H. Gao, A. Pi, F. Huang, Study on penetration characteristics of high-speed elliptical cross-sectional projectiles into concrete. *Int. J. Impact Eng.* **132**, 103311 (2019).

74. C. Seong, Tanged points, microblades and Late Palaeolithic hunting in Korea. *Antiquity* **82**, 871–883 (2008).

75. J.-P. Yue, S.-X. Yang, Y.-Q. Li, M. Storozum, Y.-M. Hou, Y. Chang, M. D. Petraglia, Human adaptations during MIS 2: Evidence from microblade industries of Northeast China. *Palaeogeogr. Palaeoclimatol. Palaeoecol.* **567**, 110286 (2021).

76. M. Zhang, Rethinking microblade technology research in northeastern Asia. *J. Paleolit. Archeol.* **4**, 17 (2021).

77. H. L. Smith, T. Goebel, Origins and spread of fluted-point technology in the Canadian ice-free corridor and eastern Beringia. *Proc. Natl. Acad. Sci. U.S.A.* **115**, 4116–4121 (2018).

78. H. L. Smith, J. T. Rasic, T. Goebel, "Biface traditions of northern Alaska and their role in the peopling of the Americas," in *Paleoamerican Odyssey*, K. E. Graf, C. V. Ketron, M. R. Waters, Eds., Center for the Study of the First Americans (Texas A&M University, College Station, 2013), pp. 105–123.

79. G. R. Scott, D. H. O'Rourke, J. A. Raff, J. C. Tackney, L. J. Hlusko, S. A. Elias, L. Bourgeon, O. Potapova, E. Pavlova, V. Pitulko, J. F. Hoffecker, Peopling the Americas: Not "out of Japan". *PaleoAmerica* **7**, 309–332 (2021).

80. E. A. Bennett, Y. Liu, Q. Fu, *Reconstructing the Human Population History of East Asia through Ancient Genomics* (Cambridge Univ. Press, 2024).

81. Y. Kudo, Calibrated radiocarbon dates of the earliest pottery in the Japanese archipelago: Distinction between IntCal04 and IntCal09, and "the year 13,000 problem". *Bull. Nat. Mus. Jpn. Hist.* **172**, 101–116 (2012).

82. K. Morisaki, N. Oda, D. Kunikita, Y. Sasaki, Y. Kuronuma, A. Iwase, T. Yamazaki, N. Ichida, H. Sato, Sedentism, pottery and inland fishing in late Glacial Japan: A reassessment of the Maedakochi site. *Antiquity* **93**, 1442–1459 (2019).

83. D. Natsuki, Migration and adaptation of Jomon people during Pleistocene/Holocene transition period in Hokkaido, Japan. *Quat. Int.* **608–609**, 49–64 (2022).

84. D. Kunikita, I. Shevkomud, K. Yoshida, S. Onuki, S. T. Yamahara, H. Matsuzaki, Dating charred remains on pottery and analyzing food habits in the Early Neolithic period in Northeast Asia. *Radiocarbon* **55**, 1334–1340 (2013).

85. K. Nagai, Flake scar patterns of Japanese tanged points: Toward an understanding of technological variability during the Incipient Jomon. *Anth. Sci.* **115**, 223–226 (2007).

86. T. Gakuhari, S. Nakagome, S. Rasmussen, M. E. Allentoft, T. Sato, T. Korneliussen, B. Ní Chuinneagáin, H. Matsumae, K. Koganebuchi, R. Schmidt, S. Mizushima, O. Kondo, N. Shigehara, M. Yoneda, R. Kimura, H. Ishida, T. Masuyama, Y. Yamada, A. Tajima, H. Shibata, A. Toyoda, T. Tsurumoto, T. Wakebe, H. Shitara, T. Hanihara, E. Willerslev, M. Sikora, H. Oota, Ancient Jomon genome sequence analysis sheds light on migration patterns of early East Asian populations. *Comm. Bio.* **3**, 437 (2020).

87. Y. Watanabe, Y. Wakiyama, D. Waku, G. Valverde, A. Tanino, Y. Nakamura, T. Suzuki, K. Koganebuchi, T. Gakuhari, T. Katsumura, M. Ogawa, A. Toyoda, S. Mizushima, T. Nagaoka, K. Hirata, M. Yoneda, T. Nishimura, M. Izuho, Y. Yamada, T. Masuyama, R. Takahashi, J. Ohashi, NCBN Controls WGS Consortium, H. Oota, Cold adaptation in Upper Paleolithic hunter-gatherers of eastern Eurasia. bioRxiv 2024.05.03.591810 [Preprint] (2024). https://doi.org/10.1101/2024.05.03.591810.

88. D. G. Duke, Haskett spear weaponry and protein-residue evidence of proboscidean hunting in the Great Salt Lake Desert, Utah. *PaleoAmerica* **1**, 109–112 (2015).

89. D. G. Duke, D. E. Stueber, "Haskett and its Clovis Parallels," in *New Perspectives on Stemmed and Fluted Technologies of the American Far West*, R. L. Rosencrance, J. E. Pratt, Eds. (Univ. of Utah Press, 2024), pp. 79–112.

90. M. B. Collins, M. Kay, *Clovis Blade Technology: A Comparative Study of the Keven Davis Cache* (Univ. of Texas Press, 2002).

91. M. B. Collins, J. Lohse, "The nature of Clovis blades and blade cores," in *Entering America: Northeast Asia and Beringia Before the Last Glacial Maximum*, D. B. Madsen, Ed. (Univ. of Utah Press, 2004), pp. 159–183.

92. D. J. Meltzer, Overkill, glacial history, and the extinction of North America's Ice Age megafauna. *Proc. Natl. Acad. Sci. U.S.A.* **117**, 28555–28563 (2020).

93. T. J. Williams, "Testing the Atlantic ice hypothesis: The blade manufacturing of Clovis, Solutrean and the broader technological aspects of production in the Upper Palaeolithic," thesis, University of Exeter (2014).

94. S. Oppenheimer, B. Bradley, D. Stanford, Solutrean hypothesis: Genetics, the mammoth in the room. *World Arch.* **46**, 752–774 (2014).

95. M. Izuho, New AMS ^{14}C dates for the Upper Paleolithic sites in the Tokachi Plain on Hokkaido (Japan). *Paleolit. Res.* **9**, 137 (2013).

96. J. Takakura, Rethinking the disappearance of microblade technology in the Terminal Pleistocene of Hokkaido, northern Japan: Looking at archaeological and palaeoenvironmental evidence. *Quaternary* **3**, 21 (2020).

97. M. Yi, X. Gao, F. Li, F. Chen, Rethinking the origin of microblade technology: A chronological and ecological perspective. *Quat. Internat.* **400**, 130–139 (2016).

98. F. Iizuka, M. Izuho, Late Upper Paleolithic–initial Jomon transitions, southern Kyushu, Japan: Regional scale to macro processes—A close look. *Quat. Int.* **441**, 102–112 (2017).

99. J. Clark, A. E. Carlson, A. V. Reyes, E. C. Carlson, L. Guillaume, G. A. Milne, L. Tarasov, M. Caffee, K. Wilcken, D. H. Rood, The age of the opening of the ice-free corridor and implications for the peopling of the Americas. *Proc. Natl. Acad. Sci. U.S.A.* **119**, e2205042119 (2022).

100. D. Froese, J. M. Young, S. L. Norris, M. Margold, Availability and viability of the ice-free corridor and Pacific coast routes for the peopling of the Americas. *SAA Archaeol. Rec.* **19**, 27–33 (2019).

101. S. K. Praetorius, J. R. Alder, A. Condron, A. C. Mix, M. H. Walczak, B. E. Caissie, J. M. Erlandson, Ice and ocean constraints on early human migrations into North America along the Pacific coast. *Proc. Nat. Acad. Sci. U.S.A.* **120**, e2208738120 (2023).

102. M. L. Steffen, New age constraints for human entry into the Americas on the north Pacific coast. *Sci. Rep.* **14**, 4291 (2024).

103. Y. Kaifu, L. Chih-Hsing, I. Nobuyuki, Y. Masahisa, A. Iwase, Y.-L. K. Chang, M. Uchida, K. Hara, K. Amemiya, Y. Sung, K. Suzuki, M. Muramatsu, M. Tanaka, S. Hanai, T. Hawira, S. Uchida, M. Fujita, Y. Miyazawa, K. Nakamura, P.-L. Wen, A. Goto, Paleolithic seafaring in East Asia: An experimental test of the dugout canoe hypothesis. *Sci. Adv.* **11**, 26 eadv5507 (2025).

104. Y.-L. K. Chang, Y. Miyazawa, X. Guo, S. Varlamov, H. Yang, Y. Kaifu, Traversing the Kuroshio: Paleolithic migration across one of the world's strongest ocean currents. *Sci. Adv.* **11**, eadv5508 (2025).

105. M. Fujita, F. Mizuno, S. Yamasaki, "Prehistoric migration of *Homo sapiens* in the Ryuku Islands," in *The Prehistory of Human Migration – Human Expansion*, *Resource Use and Mortuary Practice in Maritime Asia*, R. Ono, A. Pawlik, Eds. (IntechOpen, 2024).

106. M. F. Johnson, "Additional research at Cactus Hill, preliminary description of Northern Virginia Chapter-ASV's 1993 and 1995 excavations," in *Archaeological Investigations of Site 44SX202*, *Cactus Hill*, *Sussex County*, *Virginia*, *Appendix G*, Virginia Department of Historic Resources Research Report Series 8 (Virginia Department of Historic Resources, Richmond, 1997).

107. J. M. McAvoy, L. D. McAvoy, *Archaeological Investigations of Site 44SX202, Cactus Hill, Sussex County*, *Virginia*, Virginia Department of Historic Resources Research Report Series 8 (Virginia Department of Historic Resources, 1997).

108. J. M. McAvoy, J. C. Baker, J. K. Feathers, R. L. Hodges, L. J. McWeeney, T. R. Whyte, "Summary of research at the Cactus Hill archaeological site, 44SX202, Sussex County, Virginia," (report prepared for the National Geographic Society in compliance with the stipulations of grant #6345–98, National Geographic Society, Committee for Research and Exploration, Washington DC, 2000).

109. J. K. Feathers, E. J. Rhodes, S. Huot, J. M. McAvoy, Luminescence dating of sand deposits related to late Pleistocene human occupation at the Cactus Hill site, Virginia, USA. *Quat. Geochron.* **1**, 167–187 (2006).

110. S. J. Fiedel, "Is that all there is? The weak case for pre-Clovis occupation of Eastern North America", in *In the Eastern Fluted Point Tradition*, J. A. M. Gingerich, Ed. (University of Utah Press, 2013), pp. 333–354.

111. C. V. Haynes, Paleoindian charcoal from Meadowcroft Rockshelter: Is contamination a problem? *Amer. Antiq.* **45**, 582–587 (1980).

112. P. Goldberg, T. L. Arpin, Micromorphological analysis of sediments from Meadowcroft Rockshelter, Pennsylvania: Implications for radiocarbon dating. *J. Field Arch.* **26**, 325–342 (1999).

113. J. I. Mead, Is it really that old? A comment about the Meadowcroft Rockshelter overview. *Amer. Antiq.* **45**, 579–582 (1980).

114. J. S. McLachlan, J. S. Clark, P. S. Manos, Molecular indicators of tree migration capacity under rapid climate change. *Ecol. Soc. Am.* **86**, 2088–2098 (2005).

115. D. E. Soltis, A. B. Morris, J. S. McLachlan, P. S. Manos, P. S. Soltis, Comparative phylogeography of unglaciated eastern North America. *Molec. Ecol.* **15**, 4261–4293 (2006).

116. J. E. Morrow, S. J. Fiedel, D. L. Johnson, M. Kornfeld, M. Rutledge, W. R. Wood, Pre-Clovis in Texas? A critical assessment of the "Buttermilk Creek Complex". *J. Arch. Sci.* **39**, 3677–3682 (2012).

117. T. A. Surovell, S. A. Allaun, B. A. Crass, J. A. M. Gingerich, K. E. Graf, C. E. Holmes, R. L. Kelly, M. Kornfeld, K. E. Krasinski, M. L. Larson, S. R. Pelton, B. T. Wygal, Late date of human arrival to North America: Continental scale differences in stratigraphic integrity of pre-13,000 BP archaeological sites. *PLOS ONE* **17**, e0264092 (2022).

118. B. R. Butler, The earlier cultural remains at Cooper's Ferry. *Tebiwa* **12**, 35–50 (1969).

119. L. S. Cressman, H. Williams, A. D. Krieger, *Early Man in Oregon: Archaeological Studies in the Northern Great Basin*, University of Oregon Monographs, Studies in Anthropology 3 (Eugene, 1940).

120. L. S. Cressman, F. C. Baker, H. P. Hansen, P. Conger, R. F. Heizer, *Archaeological Researches in the Northern Great Basin* (Carnegie Institution of Washington, 1942).

121. D. L. Jenkins, "Distribution and dating of cultural and paleontological remains at the paisley five mile point caves in the Northern Great Basin," in *Paleoindian or Paleoarchaic: Great Basin Human Ecology at the Pleistocene-Holocene Transition*, K. Graf, D. Schmidt, Eds. (Univ. of Utah Press, 2007), pp. 57–81.

122. L.-M. Shillito, H. L. Whelton, J. C. Blong, D. L. Jenkins, T. J. Connolly, I. D. Bull, Pre-Clovis occupation of the Americas identified by human fecal biomarkers in coprolites from Paisley Caves, Oregon. *Sci. Adv.* **6**, eaba6404 (2020).
